# CD73 contributes to anti‐inflammatory properties of afferent lymphatic endothelial cells in humans and mice

**DOI:** 10.1002/eji.201948432

**Published:** 2020-10-29

**Authors:** Dominik Eichin, Alberto Pessia, Akira Takeda, Joni Laakkonen, Lydia Bellmann, Matti Kankainen, Beat A. Imhof, Patrizia Stoitzner, Jing Tang, Marko Salmi, Sirpa Jalkanen

**Affiliations:** ^1^ MediCity Research Laboratory University of Turku Turku Finland; ^2^ Research Program in Systems Oncology Faculty of Medicine University of Helsinki Helsinki Finland; ^3^ Department of Dermatology, Venereology & Allergology Medical University of Innsbruck Innsbruck Austria; ^4^ Medical and Clinical Genetics University of Helsinki and Helsinki University Hospital Helsinki Finland; ^5^ Hematology Research Unit Helsinki University of Helsinki Helsinki Finland; ^6^ Translational Immunology Program University of Helsinki Helsinki Finland; ^7^ Department of Pathology and Immunology, Centre Médical Universitaire (CMU), Medical Faculty University of Geneva Geneva Switzerland; ^8^ Institute of Biomedicine University of Turku Turku Finland

**Keywords:** CD73, dendritic cells, lymphatic endothelial cells, siRNA, vascular biology

## Abstract

CD73 is an important ectoenzyme responsible for the production of extracellular adenosine. It is involved in regulating inflammatory responses and cell migration and is overexpressed in various cancers. The functions of CD73 in blood endothelial cells are understood in detail, but its role on afferent lymphatics remains unknown. Moreover, anti‐CD73 antibodies are now used in multiple clinical cancer trials, but their effects on different endothelial cell types have not been studied. This study reveals that a previously unknown role of CD73 on afferent lymphatics is to dampen immune responses. Knocking it out or suppressing it by siRNA leads to the upregulation of inflammation‐associated genes on lymphatic endothelial cells and a more pro‐inflammatory phenotype of interacting dendritic cells in vitro and in vivo. In striking contrast, anti‐CD73 antibodies had only negligible effects on the gene expression of lymphatic‐ and blood‐endothelial cells. Our data thus reveal new functions of lymphatic CD73 and indicate a low likelihood of endothelial cell–related adverse effects by CD73 targeting therapeutic antibodies.

## Graphical abstract

Silencing CD73 increases the expression of MHCI and ICAM‐1 on lymphatic endothelial cells (LECs), resulting in elevated MHCII and CD83 levels on DCs. This is also the case for inflammatory genes such as TGFB1 or CD69. In contrast, modulating CD73 expression with antibodies showed no such effect with LECs or blood endothelial cells.

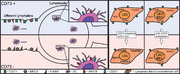

## Introduction

CD73 (ecto‐5′‐nucleotidase), a GPI‐anchored ectoenzyme, dephosphorylates AMP into adenosine. This generates an anti‐inflammatory and pro‐angiogenic halo that can reduce immune activation and promote cancer progression [1, 2]. Due to its abundant expression on many types of tissues and cells throughout the body, CD73 is one of the most important producers of extracellular adenosine. Once produced, adenosine acts by engaging a variety of different adenosine receptors (A1, A2A, A2B, and A3) on the cell surface. The exact outcome of this interaction depends not only on the engaged receptor, but also on the cellular partner [3, 4, 5]. As some cells express CD73 as well as adenosine receptors concomitantly, even self‐regulating feedback loops are possible [6, 7].

CD73 is expressed on cells of the immune system such as B‐ and T‐lymphocytes, but also blood endothelial cells (BECs) and lymphatic endothelial cells (LECs) and even certain cancer cells are CD73 positive [8, 9]. On most of these cells, CD73 exerts immune system regulating roles [10]. In cancer, for example, CD73 is limiting the activity of the immune system and thereby prevents the clearance of cancer cells. To tackle this, several companies are developing CD73 targeting antibodies that have been shown to stimulate the immune response [11, 12, 13]. However, their systemic effects on other nontargeted cells and tissues, such as the vasculature, have not been thoroughly investigated and what role CD73 plays in those tissues is understudied. One example is LECs, where the role of CD73 is unknown. This is especially noteworthy, as while the efferent side does not express CD73, the afferent side of the lymphatic network, from the periphery to the draining LN, is highly CD73 positive [14].

Earlier attempts to shed light into the function of afferent lymphatic CD73 did not find a specific role. However, they revealed that the importance of CD73 is significantly different from the role it exerts on BECs. Unlike in BECs, in LECs it does not affect leukocyte migration, angiogenesis, or permeability, and therefore its function remains unknown [14, 15]. In the light of ongoing clinical cancer trials that are utilizing systemically administered CD73 modifiers such as inhibitors or blocking antibodies (Supporting Information Table S1) to boost the immune system, it therefore becomes imperative to decipher this function and prevent potential deleterious effects that could be caused by a systemic reduction of CD73 activity in the lymphatic vasculature. We therefore use, for the first time, unbiased genome‐wide analysis to investigate what role CD73 plays on LECs and what effects its modification with different treatment modalities can have on components of the vascular system.

## Results

### CD73 silencing in LECs results in a pro‐inflammatory phenotype

**Figure 1:**
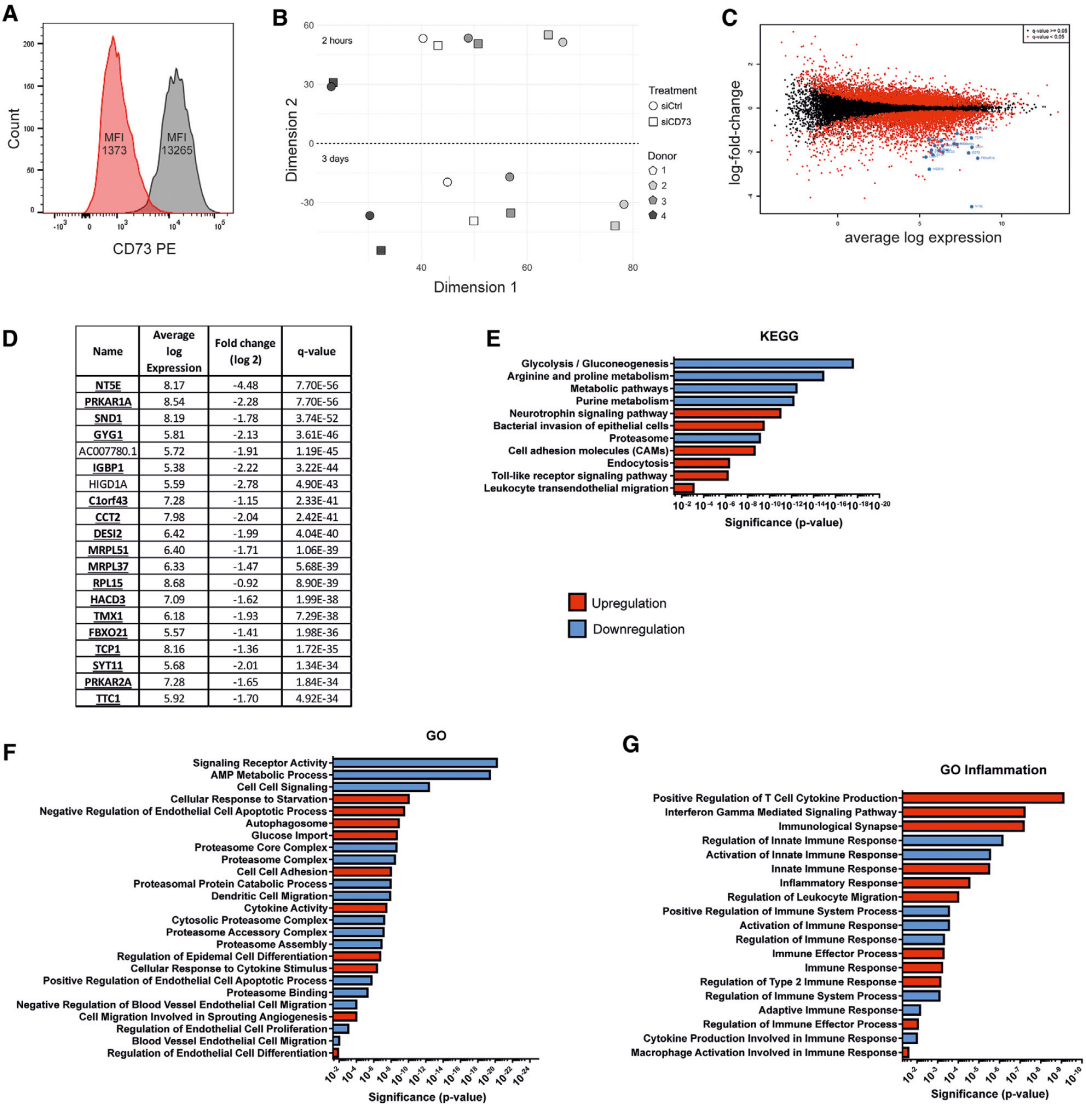
siCD73 treated primary lymphatic endothelial cells (LECs) show extensive transcriptional changes and altered pro‐inflammatory pathways. (A) Representative histogram from flow‐cytometry showing reduction of CD73 after siRNA treatment. CD73‐silenced LECs are depicted with red (MFI: 1373), nontargeting control‐silenced cells in gray (MFI: 13265). (B) Multidimensional scaling (MDS) plot of RNA‐sequencing after siRNA treatment, built from Euclidian distances of logCPM values from sorted cells of four different HDMEC donors. Observations cluster according to donor, time, and treatment. (C) Limma‐plot indicating significantly changed genes after CD73‐silencing in red. The 20 most significantly altered genes are encircled. (D) Table depicting the 20 most significantly altered genes from (C) with their global average expression (logCPM), fold change, and *q*‐value. Genes in bold were altered similarly after siRNA and CRISPR/Cas9. (E and F) Pathway analysis results using the KEGG and GO database with significant up‐ and downregulations following silencing. (G) Inflammation pathways obtained using the GO database with significant up‐ and downregulations. Data are obtained from one experiment with four different biological donors as described in more detail in the method section.

To determine the role of CD73 in afferent lymphatics, we silenced the expression of CD73 in primary human LECs with a pool of four siRNA constructs, CRISPR/Cas9, or a single siRNA. Three days after silencing with the siRNA pool, the gene expression of CD73 was on average reduced by more than 96% (76% after CRISPR/Cas9; 98% after single siRNA), while the protein expression went down by 88% (95% after CRISPR/Cas9; 96% after single siRNA; Fig. 1A and Supporting Information Fig. S1A and B; gating is shown in Supporting Information Fig. S2). The siRNA‐mediated knockdown of CD73 also led to efficient inhibition of ecto‐5′‐nucleotidase (CD73) activity in LECs, but did not interfere with ATPase, ADPase, or adenylate kinase activities (Supporting Information Fig. S1C), suggesting that there were no off‐target effects on the extracellular ATP metabolism.

To better understand the changes caused by modifying CD73 on the genetic level, we performed RNA sequencing of primary HDMEC‐derived siRNA‐pool silenced LECs after 2 h and 3 days of silencing. The results revealed that the cells clustered most strongly according to time point, followed by donor and treatment (Fig. 1B). As expected, silenced samples from the 3‐day time point differed much more from their nontargeted siRNA‐treated controls than samples from the 2‐h time point.

Based on DESeq and limma, no genes were significantly altered 2 h after silencing, while after 3 days, 7536 genes showed a significant alteration as depicted in red in the limma plot (Fig. 1C). The 20 most significantly altered genes are encircled in Fig. 1C and listed in Fig. 1D.

To understand the possible consequences of the observed gene alterations, we conducted pathway analysis using the Kyoto Encyclopedia of Genes and Genomes (KEGG) and Gene Ontology (GO) enrichment analysis (Fig. 1E and F). Silencing caused a profound change in a multitude of pathways such as glycolysis, purine metabolism, cell–cell signaling, or cytokine activity. Intriguingly, also a plethora of inflammation‐related pathways was altered to a pro‐inflammatory direction (Fig. 1G). Among these pathways were, for example, the “Interferon Gamma Mediated Signaling Pathway,” “Regulation of Innate Immune Response,” and the “Inflammatory Response.”

### Inflammation associated genes are upregulated following CD73 silencing

**Figure 2:**
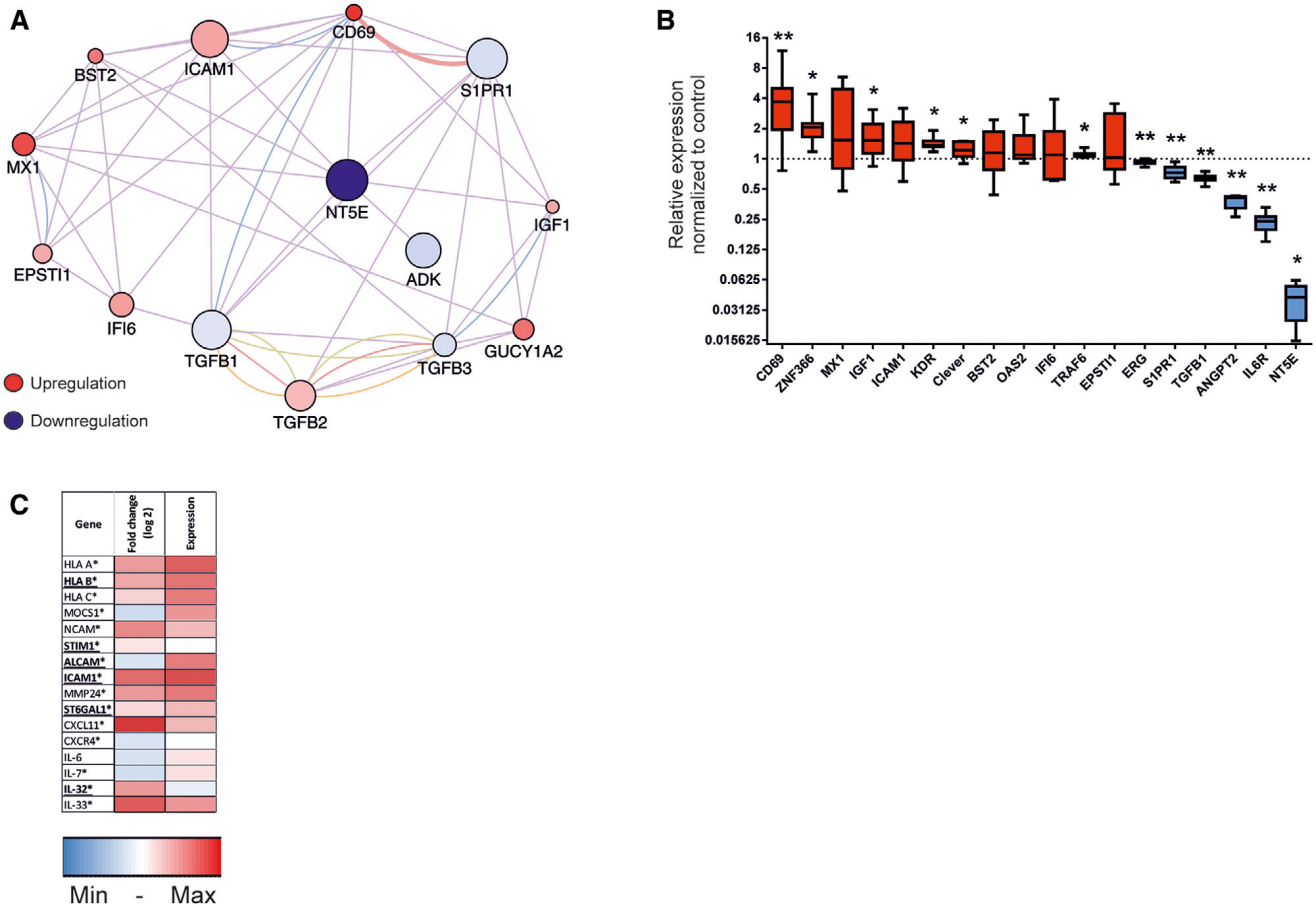
Inflammation‐ and DC‐associated genes are altered after siCD73 treatment of LECs. (A) Interaction networks of CD73 (= NT5E) and inflammation‐associated genes obtained with CytoScape of CD73‐siRNA silenced cells compared to their nontargeted siRNA‐treated controls. The circle size indicates the expression of the gene, the color of its up‐ or downregulation (red and blue, respectively), ranging from +9.58 to −22.32. The color of the connectors indicate co‐expression (purple), co‐localization (blue), physical interaction (pink), predicted interaction (orange), or a shared protein domain (green). (B) qPCR verification of important RNA‐seq hits (CD69, ZNF366, MX1, IGF1, ICAM1, KDR, Clever, BST2, OAS2, IFI6, TRAF6, EPSTI1, ERG, S1PR1, TGFB1, ANGPT2, IL6R, NT5E) shown as fold changes of siCD73‐treated LECs compared and normalized to the nontargeted control, analyzed with Wilcoxon matched‐pairs signed rank test. Data are depicted as boxplots showing the median with Min and Max values as whiskers. (C) Altered genes after siCD73 treatment that code for proteins shown to interact with DCs, their up‐ or downregulation and expression are shown. Genes in bold were altered similarly after pool‐siRNA and CRISPR/Cas9. *p < 0.05, **p < 0.01. In (A) and (C), data are from one experiment with four different biological donors; in (B), the data are from three independent experiments with four, two, and three different biological donors, except for the genes ZNF366, OAS2, KDR, and TRAF6 where the data are from two independent experiments with four and three biological donors.

When having a closer look at known inflammatory genes and their network, we observed a predominance of upregulated genes, indicating an elevated inflammatory phenotype after CD73 silencing (Fig. 2A).

To verify the results of our RNA sequencing, we tested some of the most differentially expressed genes as well as several known inflammatory genes such as CD69, MX1, TRAF6, and TGFB1 for their expression levels with qPCR. The results shown in Fig. 2B confirmed the results obtained in the RNA sequencing analysis, demonstrating that silencing CD73 affects a multitude of (inflammatory) genes.

To limit the chance of detecting alterations due to off‐target effects, we additionally performed sequencing on CRISPR/Cas9 CD73KO cells. As the silencing on the gene level was not as effective as with siRNA, single‐cell RNA sequencing was performed in order to focus on cells with efficient CD73 knockdown. Overall, the smaller reduction in CD73 gene expression compared to siRNA resulted in less and smaller alterations detectable in other genes. Nevertheless, around 80% of genes that we looked at in more detail (listed in Figs. 1 and 2) showed regulation to the same direction (i.e., upregulation or downregulation) as with siRNA (e.g., HLA‐B, ICAM1, S1PR1, or TGFB) in cells of both analyzed individuals. Furthermore, we also found the same tendency when we looked at selected genes with qPCR following CD73 silencing with a single siRNA (Supporting Information Fig. S3).

**Figure 3:**
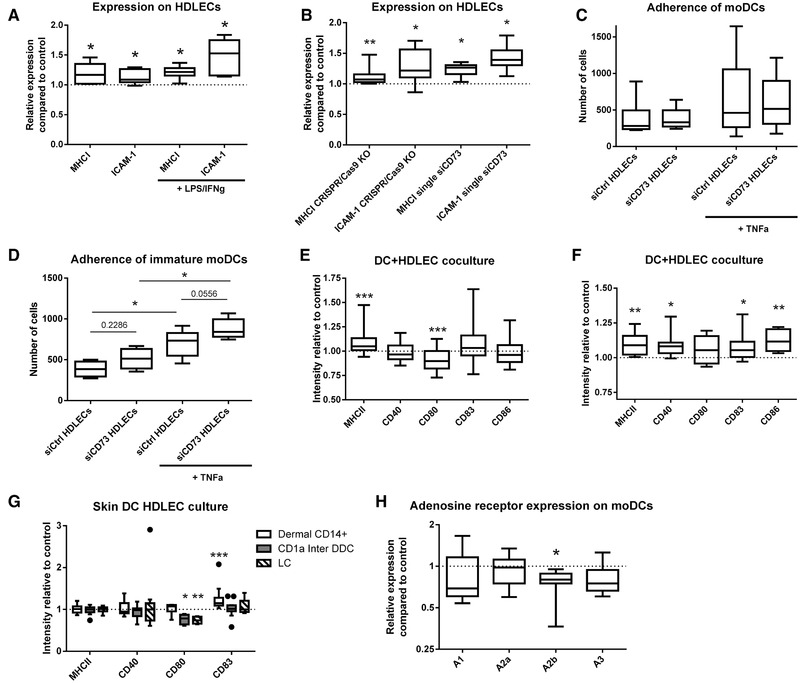
CD73‐silenced LECs promote an inflammatory phenotype on dendritic cells. (A) Relative expression of MHCI and ICAM‐1 on LECs after pool‐siCD73 treatment and LPS/IFN‐γ exposure as determined with flow cytometry compared to nontargeted siRNA‐treated controls. The data are from two to three independent experiments with two, four, and one different biological donor(s). (B) Relative expression of MHCI and ICAM‐1 on LECs after CRISPR/Cas9 and single‐siCD73 treatment with LPS/IFN‐γ exposure compared to controls determined with flow cytometry. Data are from three to four independent experiments with two to three biological donors. (C) Adherence of moDCs to CD73‐silenced and nontargeted control LECs in the steady‐state and after TNF‐α treatment of LECs. The data are from four independent experiments with one, two, two, and two (one, two, one, one after TNF‐α) different biological donors (one, two, one, and one donor(s) after TNF‐α). (D) As in (C), but with immature moDCs. Data are from two independent experiments with two to three donors. (E) Relative expression of moDC maturation markers following co‐culture with siCD73 treated LECs and their nontargeted controls measured by flow cytometry. The data are from 17 independent experiments with one to two different biological donors. (F) As in (E), but CD73 on LECs has been knocked out with CRISPR/Cas9. Data are from three independent experiments with two to three donors. (G) Relative expression of skin‐DC maturation markers following co‐culture with pool‐siCD73 treated LECs and their nontargeted controls measured by flow cytometry. The data are from six independent experiments with one to three different biological donors (*n* = 7–11). DDC = dermal dendritic cells; LC = Langerhans cells. (H) Relative adenosine receptors (A1, A2a, A2b, A3) expression on DCs following co‐culture with pool‐siCD73‐treated LECs compared to nontargeted control measured by qPCR. The data are from four independent experiments with one, two, two, and two different biological donors. Data have been analyzed with Wilcoxon matched‐pairs signed rank test. Data are depicted as boxplots showing the median with Min and Max values as whiskers. *p < 0.05, **p < 0.01, ***p < 0.001.

To narrow down possible functional effects that the knockdown of CD73 might have, we investigated interaction partners of LECs that would be influenced by this apparent cell modification. One of the most important cell types to interact with LECs are dendritic cells (DCs). It was therefore intriguing to see that a number of genes coding for receptors, adhesion molecules, and chemokines, which have a potential partner on DCs, had been altered on LECs by CD73‐silencing. The extent of this alteration (as well as the overall gene expression levels) is depicted in Fig. 2C, which among others shows a clear upregulation of several HLA molecules (HLA‐A, fold change of “1.36”; HLA‐B, fold change of “1.95”; HLA‐C, fold change of “1.81”) and ICAM‐1 (fold change of “2.76”), as well as a reduction of IL‐7 (fold change “0.71”) and MMP24 (fold change of “0.51”) following the silencing of CD73 using the siRNA pool. Additionally, we verified the increased expression of HLA and ICAM‐1 at the protein level following CD73 pool‐siRNA treatment (Fig. 3A and Supporting Information Fig. S4A). This increase could also be detected on inflamed LECs (Fig. 3A and Supporting Information Fig. S4B and C) that have elevated levels of CD73 (Supporting Information Fig. S5A) as well as on CRISPR/Cas9 KO and single siRNA silenced cells (Fig. 3B). Interestingly, despite the changes in CD73 following inflammation, only ICAM‐1 showed a more pronounced protein expression after LPS/IFN‐γ exposure in CD73‐silenced cells, while the relative values of the MHC class I molecules remained constant. Overall, the pattern of these changes demonstrates a more pro‐inflammatory LEC phenotype and an altered interaction between LECs and DCs.

### Lymphatic CD73 alters DC maturation in a contact‐dependent manner

As the CD73‐regulated transcriptome in LECs could alter the physical interaction between LECs and DCs or the maturation status of DCs, we investigated this further. To test if ICAM‐1 induction in CD73‐silenced LECs would affect their interaction with DCs, we measured the adherence of 6‐h matured and immature monocyte‐derived DCs (moDCs) to LECs. There was no difference in mature moDC adherence to CD73‐silenced and control endothelial cells under resting conditions or after TNF‐α induction (Fig. 3C). However, immature moDCs bound significantly more to TNF‐α‐treated LECs and there was a strong trend for increased binding to silenced LECs under these conditions (Fig. 3D).

To study the potential effect of LEC‐CD73 on DC maturation, we co‐cultured partly matured (6 h with LPS/IFN‐γ) moDCs with CD73‐silenced LECs, CRISPR/Cas9 KO LECs, or their respective controls. While these DCs had elevated adenylate kinase and ADPase activity, no AMPase (= CD73) activity could be detected (Supporting Information Fig. S5B), verifying our flow‐cytometric result that did not show any CD73 protein expression on the surface of immature and mature moDCs.

After 1 day of co‐culture, the DCs showed an increase in MHCII and CD83 in both cultures, while only culturing them with CRISPR/Cas9 KO cells increased CD40 and CD86. In addition, only exposure to siCD73‐treated LECs lowered the expression of CD80 (Fig. 3E and F and Supporting Information Fig. S6A and B; gating is depicted in Supporting Information Fig. S7). These findings are consistent with the pro‐inflammatory profile of CD73‐silenced LECs. Similar activation of human skin‐derived DCs (dermal DCs and Langerhans cells (LC); gating strategy depicted in Supporting Information Fig. S8) was observed after co‐culturing these primary cells with CD73‐silenced LECs (Fig. 3G and Supporting Information Fig. S9). Moreover, co‐culture with CD73‐deficient LECs led to downregulation of moDC A2b adenosine receptor gene expression, while also showing a potential reduction in A1 and A3 receptors (Fig. 3H).

**Figure 4:**
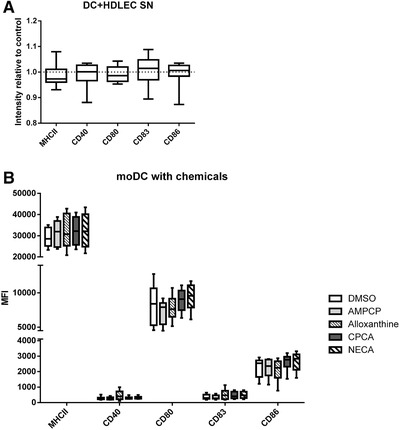
Physical contact, rather than secreted factors from LECs, is responsible for the changed inflammatory profile. (A) Relative expression of moDC maturation‐markers following culture with supernatants from pool‐siCD73‐treated LECs and their nontargeted control. The data are from seven independent experiments with one to two different biological donors (*n* = 10 biological replicas, Wilcoxon matched‐pairs signed rank test). (B) Median fluorescent intensity values of moDC maturation markers after culture with compounds affecting the adenosine pathway. The data are from five to seven independent experiments with one biological donor. Data are depicted as boxplots showing the median with Min and Max values as whiskers and measured by flow cytometry. AMPCP (adenosine 5′‐(α,β‐methylene)diphosphate sodium salt; CD73 inhibitor); alloxanthine (inihibitor of xhantine oxidase); CPCA (5′‐(N‐cyclopropyl)carboxamidoadenosine; A_2_‐adenosine receptor agonist); NECA (5′‐(N‐ethylcarboxamido)adenosine; adenosine receptor agonist).

Next, we exposed DCs to the supernatant harvested from CD73‐silenced and control LECs to determine if soluble mediators or cell–cell interactions caused the observed inhibitory effects. We found no difference in the maturation status of the DCs (Fig. 4A; gating shown in Supporting Information Fig. S10), arguing against a soluble mediator affecting the DC phenotype. This was confirmed by our results from experiments, where we exposed DCs to various chemical compounds affecting different parts of the enzymatic cascade regulating the dephosphorylation of AMP to adenosine (Fig. 4B). In these experiments, we also observed that the maturation state remained unaffected by these modulations of the enzymatic cascade. Collectively, these data support the idea that cell–cell contact is needed for altering DC maturation by CD73‐silenced LECs, and furthermore show that moDC activation is resistant against soluble modulators of the enzymatic cascade.

Overall, these data suggest that while CD73 on LECs is not directly involved in DC adhesion, it limits their pro‐inflammatory maturation in a contact‐dependent manner.

### More inflammatory DCs and lymphatic vessels in CD73^−/−^ mice after immunological challenge

**Figure 5:**
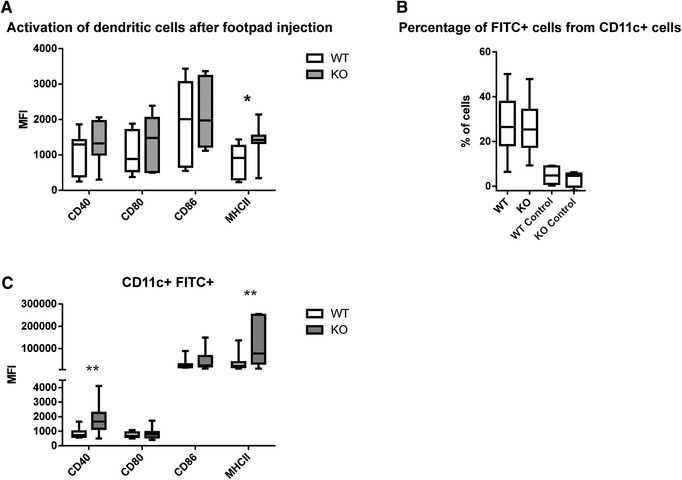
CD73 KO animals show elevated maturation of DCs after an inflammatory stimulus. (A) Expression of DC maturation markers following footpad injection of OVA and incomplete Freud's adjuvant in CD73 WT and KO animals. The data are from two independent experiments with total of four different animals per group. (B) Percentage of dendritic cells is positive for FITC in the draining LN or control LN after FITC ear painting. (C) Expression of DC maturation markers after FITC ear painting on FITC positive cells in CD73 WT and KO animals. In (B) and (C), the data are from four different experiments with total of seven different animals per group. Data are depicted as boxplots showing the median with Min and Max values as whiskers and measured by flow cytometry. Data are analyzed with Wilcoxon matched‐pairs signed rank test. *p < 0.05, **p < 0.01.

To assess if the same phenotype can be found in vivo, we utilized CD73‐deficient mice in two different experimental approaches. In the first approach, we induced inflammation by injecting ovalbumin and incomplete Freund's adjuvant into the footpad of CD73‐deficient and wild‐type (WT) animals. When analyzing the maturation status of DCs obtained from the draining LNs, we could observe significantly elevated levels of MHCII and moderate increases in CD40 and CD80 (Fig. 5A).

In the second approach, we painted the ears of mice from both genotypes with an irritant FITC solution. After 2 days, we harvested DCs from the draining LNs and compared the number of FITC‐positive cells as well as their activation status between KO and WT animals. While a comparable percentage of DCs were FITC positive (Fig. 5B), thereby representing cells that had taken up FITC in the periphery and migrated to the LN, there were differences in the activation levels of these cells (Fig. 5C). Similar to our other results, CD73 deficiency led to an increased maturation of DCs as seen by elevated levels of MHCII and CD40 (Fig. 5C).

In addition, we determined ICAM‐1 levels on lymphatic vessels from WT and KO animals. This was done in steady state and following challenge with oxazolone and revealed a trend toward elevated ICAM‐1 levels in KO animals under both conditions (Supporting Information Fig. S11).

### Anti‐CD73 antibodies inhibit the ectonucleotidase function via distinct mechanisms

**Figure 6:**
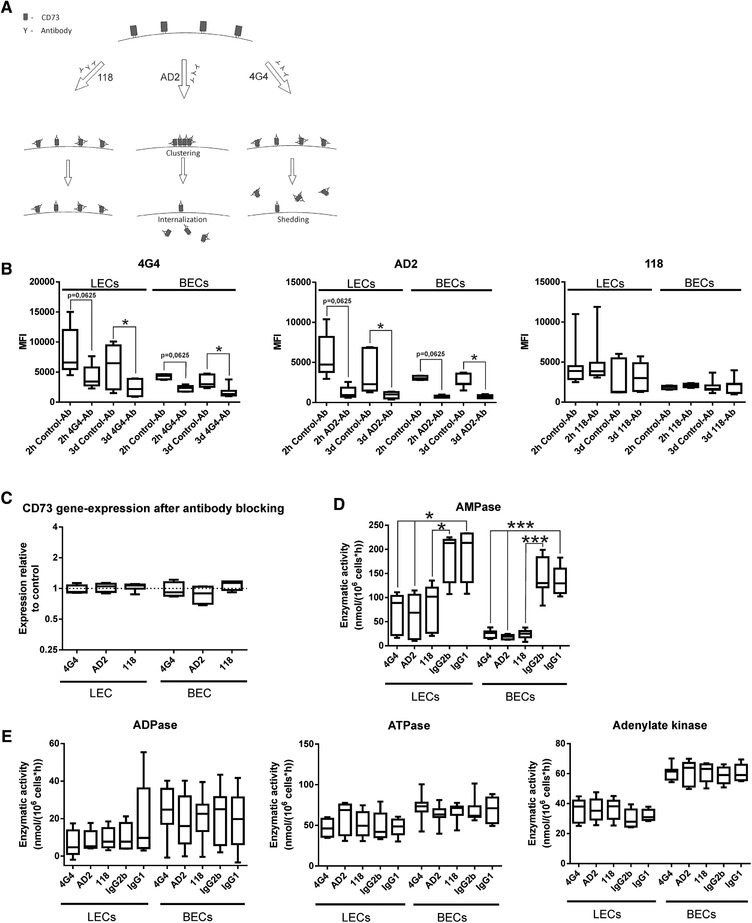
CD73‐targeting antibodies alter its expression on LECs and BECs in an epitope‐specific manner. (A) Graphical overview of the different modus operandi of CD73 antibodies 118, AD2, and 4G4. (B) CD73 expression determined by flow‐cytometry on LECs and BECs following blocking and staining with CD73 antibodies 4G4, AD2, or 118, respectively. The data are from three (2‐h time point) to four (3 days time point) independent experiments with one to three different biological donors (*n* = 5–8). (C) CD73 (NT5E) gene expression determined by qPCR following blocking with CD73‐specific antibodies on LEC and BEC cells compared to control‐antibody–treated cells. The data are from two to three independent experiments with two to three different biological donors (*n* = 4–7). (D) Enzymatic activity of AMPase (CD73), following blocking with CD73‐specific antibodies on LEC and BEC cells and was analyzed by scintillation β‐counting. The data are from two to three independent experiments with two to four different biological donors (*n* = 5–7), analyzed by Mann–Whitney *U* test. (E) Enzymatic activity of ADPase, ATPase, and adenylate kinase following blocking with CD73‐specific antibodies on LEC and BEC cells and was analyzed by scintillation β‐counting. The data are from two to three independent experiments with two to four different biological donors (*n* = 5–7), analyzed by Mann–Whitney *U* test. Data are depicted as boxplots showing the median with Min and Max values as whiskers. *p < 0.05, ***p < 0.001.

As clinical treatments are heavily relying on the use of antibodies rather than siRNA constructs, we determined the effect of the treatment of LECs with different CD73‐specific antibodies. As such treatments are often given intravenously, we additionally verified their effects on BECs to discover potential side effects. For this, three different epitope‐specific antibodies or their respective controls were applied to lymphatic‐ and blood‐endothelial cell cultures. In order to investigate early‐ and mid‐phase effects, the antibody treatment was either performed for the duration of 2 h or 3 days. We observed clear differences in terms of CD73 expression and activity between the three antibodies applied to LECs and BECs (Fig. 6A). A clear reduction of CD73 protein expression on both endothelial cell types could be observed following treatment with the antibodies AD2 and 4G4, while treatment with the 118 antibody did not alter CD73 expression (Fig. 6B). Interestingly, this change of CD73 on the protein level did not affect genomic CD73 expression (Fig. 6C). To verify whether blocking and subsequent reduction of CD73 protein would have functional effects, we investigated the enzymatic activity of CD73. We found that all of the CD73‐specific antibodies reduced the turnover rate by which AMP is dephosphorylated into adenosine (Fig. 6D), whereby no difference could be observed for ATPase, ADPase, or AK activity (Fig. 6E).

### Antibody blocking of CD73 has negligible effects on other genes and co‐cultured DCs

**Figure 7:**
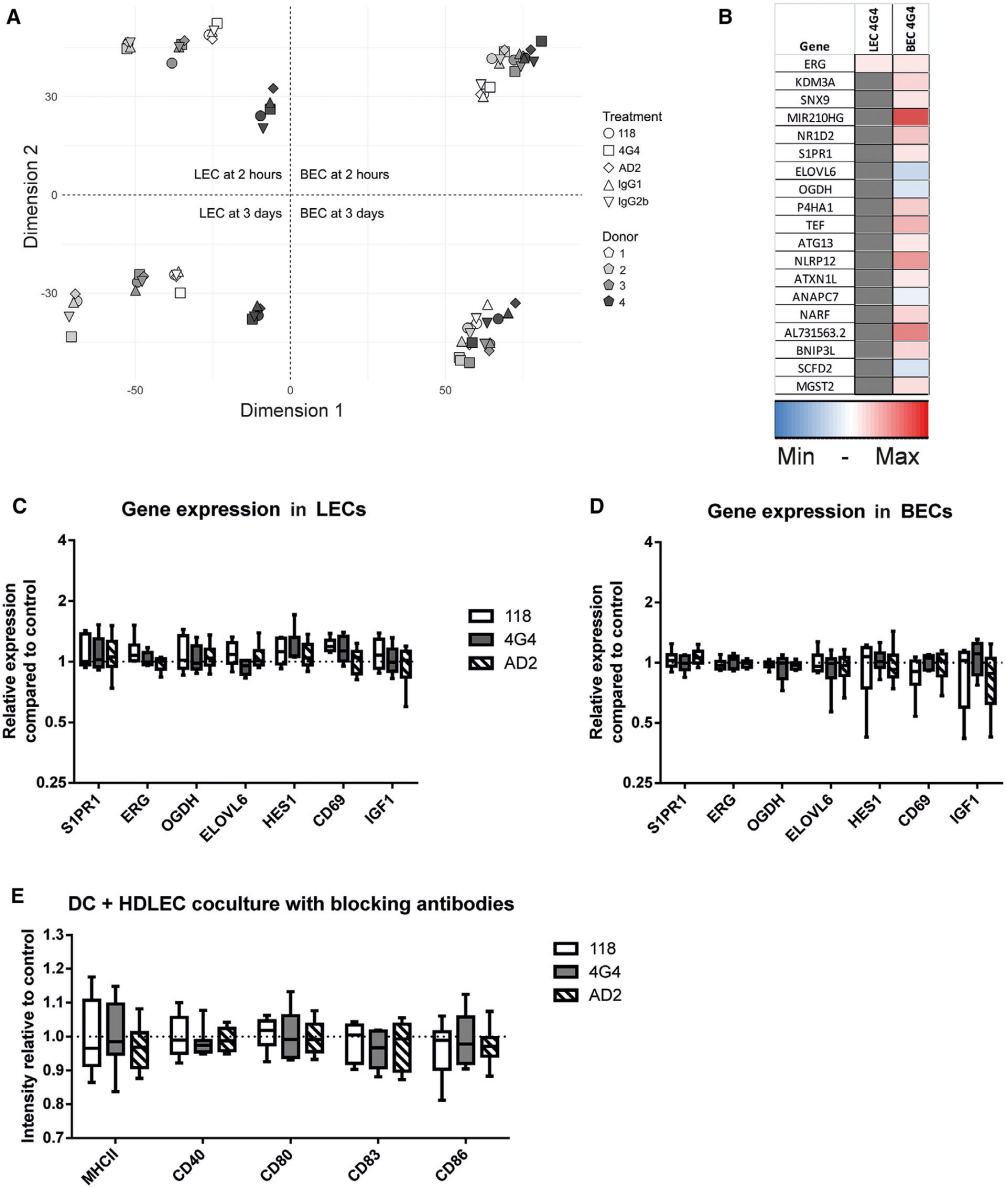
CD73‐targeting antibodies do not have significant transcriptomics effects. (A) Multidimensional scaling (MDS) plot of RNA‐sequencing after CD73‐antibody treatment, built from Euclidian distances of logCPM values. Observations cluster according to donor, time, and treatment in both LEC and BEC cells. Cells from four different sorted HDMEC donors were used. (B) Altered genes after 4G4 treatment on LEC and BEC cells are shown. Gray indicates no alteration. (C) qPCR verification of RNA‐seq hits (S1PR1, ERG, OGDH, ELOVL6, HES1, CD69, and IGF1) with LECs after antibody treatment shown as fold changes compared to control‐antibody–treated cells. The data are from two independent experiments with three different biological donors and depicted as boxplots showing the median with Min and Max values as whiskers; analyzed with Wilcoxon matched‐pairs signed rank test (nonsignificant). (D) As in (C), but obtained from BECs. The data are from two independent experiments with three different biological donors. (E) Relative expression of moDC maturation markers following co‐culture with CD73‐antibody‐treated LECs and their controls and measured by flow cytometry. The data are from three independent experiments with one to three different biological donors (*n* = 6), analyzed with Wilcoxon matched‐pairs signed rank test (nonsignificant). Data are depicted as boxplots showing the median with Min and Max values as whiskers.

To determine if targeting CD73 with antibodies has a similar impact on the endothelial transcriptome as found with CD73 silencing, we performed RNA sequencing from endothelial cells that had been incubated with each of the three antibodies. The results revealed clear gene clustering according to the antibody incubation time and the type of endothelial cells, while the outcome of the treatment with the different antibodies was similar (Fig. 7A). Interestingly, the LEC population had a bigger diversity among the donors compared to the relatively homogeneous BECs.

While there were no marked changes in mRNA levels after the 2‐h period, even after 3 days only treatment with the 4G4 antibody caused changes in one LEC gene and 19 BEC genes (Fig. 7B). As the calculated *q* values of those hits were rather big in comparison to the *q* values obtained with the siRNA silencing (10^−3^ vs. 10^−30^) and additionally also the average fold changes were small in comparison with the silencing (e.g., ERG: 1.01 vs. 9.6; S1PR1: 1.01 vs. 0.69). It was therefore not surprising that those hits could not be validated in subsequent qPCR analyses (Fig. 7C and D). Finally, we wanted to determine if modifying CD73 with the targeting antibodies would result in a similar change of phenotype of co‐cultured DCs as observed after siRNA silencing. We therefore co‐cultured moDCs and antibody‐treated LECs and compared the maturation status of the moDCs. No differences in the maturation were found regardless of the used antibody (Fig. 7E), thus being in line with the observed minor effects at the mRNA level. Collectively, the downmodulation of CD73 by antibodies has only minimal or no effects when compared to siRNA silencing and is therefore negligible for LECs and BECs.

## Discussion

We investigated the previously unknown role of CD73 on afferent lymphatics and report here that the silencing of CD73 in LECs not only has extensive consequences for the overall gene expression pattern of these cells, but also triggers immune stimulating effects on DCs in their proximity. Furthermore, no change in the gene expression pattern could be observed after blocking and/or removing CD73 with epitope‐specific antibodies on LEC or BEC cells. These results reveal an immune‐dampening role of CD73 on afferent lymphatics, while additionally providing evidence that antibody‐based treatment modalities targeting CD73 do not have major transcriptomic consequences.

By using siRNA‐ and CRISPR/Cas9‐based silencing in combination with RNA and single‐cell sequencing, we were able to examine the effects of CD73 alteration on a genome‐wide scale. This allowed us to detect an increase in inflammation‐associated genes and pathways following CD73 silencing. These results are intriguing as they, for the first time, reveal a specific role of CD73 on afferent lymphatics.

It has been shown earlier that CD73, also through the production of adenosine, can have highly anti‐inflammatory functions on several cell types [16, 17, 18]. Likewise, it also has been shown that the direct and close interaction of DCs with intestinal epithelial cells or LECs can lead to a reduced expression of the maturation marker CD86 on DCs and therefore to a less inflammatory phenotype [19, 20]. However, besides this clear logical connection, earlier studies on CD73 have exclusively focused on migratory‐ and permeability‐associated effects in both the lymphatic and the blood vasculature and neglected the inflammatory aspect [8, 14, 15, 21].

In our study, we found that CD73‐silencing caused substantial changes in a multitude of genes and pathways, including some genes that have been associated in earlier studies with CD73 [22, 23, 24]. The striking pattern of elevated pro‐inflammatory genes and pathways demonstrates that the CD73 knockdown cells express a more inflammatory profile, a finding that we could confirm on the protein level by measuring MHC class I and ICAM‐1 levels (Fig. 3A). Interestingly though, while in LECs a reduced CD73 expression led to an increase of ICAM‐1 (Fig. 3A), similarly as in HUVECs [25], ICAM‐1 levels remained the same in high endothelial venules and even decreased in carotid arteries, indicating tissue‐specific effects [15, 26]. Although ICAM‐1 has been recognized as a molecule that is elevated in an inflammatory environment and is involved in cell adhesion, we did not observe alterations in adherence of partly matured DCs to resting or inflamed CD73‐silenced LECs. However, we could observe elevated binding of immature moDCs, a finding in line with Podgrabinska et al. who reported a stronger and TNF‐α affected binding of these cells to LECs [20]. This difference between the moDC phenotypes is interesting, as in the mouse, the binding of DCs seems to be integrin independent [27]. Furthermore, we found highly significant changes in MHC class II (upregulation), CD40 (upregulation), CD83 (upregulation), CD86 (upregulation), CD80 (downregulation), as well as in the adenosine receptor A2b (downregulation) on DCs after they had been co‐cultured with siCD73‐silenced or CRISPR/Cas9 KO LECs (Fig. 3E, F, and H). In line with these findings, DCs obtained from in vivo experiments with CD73‐deficient mice also showed an increased maturation status compared to their WT controls (Fig. 5A and C) and a trend toward higher ICAM‐1 levels. This is evidence for a more pro‐inflammatory phenotype, supporting our RNA‐sequencing results, as MHC class II, CD40, CD83, and CD86 are hallmarks of mature/inflammatory DCs [20, 28, 29] and the adenosine A2b receptor has been shown to impair DC function [30]. Moreover, a reduction of the A2b receptor on DCs amplifies the CD73‐silencing effect on LECs as this receptor is crucial for mediating the effects of adenosine on DCs [31]. While we did not observe an altered moDC maturation following exposure to the LEC supernatant, this could merely be a matter of concentration dependency as mediators such as adenosine have a rather short half‐life [32]. Nevertheless, as also other modulators of the enzymatic cascade such as the adenosine analog NECA did not affect the maturation of moDCs in our setup, a physical contact seems to be required. Furthermore, CD80, while often regarded as an inflammatory molecule, has been shown to mediate inhibitory effects on T cells following DC–T cell interaction [33]. Additionally, the absence of CD80 reduces the amount of suppressive regulatory T cells [34]. In our study, DCs originating from different sources varied in the magnitude of their maturation response. While this is certainly noteworthy, these variations likely originate from the different origin (moDCs vs. skin DCs) as well as the processing and handling of those cells (moDC maturation vs. skin‐digest extraction) [35]. Potential effects on skin DCs may have been attenuated by (1) the fact that these DCs had not been enriched to purity and (2) by the fact that DCs/LCs start to mature spontaneously after enzymatic isolation from the skin, especially in unenriched suspensions [36].

In contrast to the siRNA or CRIPSR/Cas9‐treated cells, we did not observe comparable effects on the transcriptome following the treatment with CD73‐specific antibodies. This was regardless of the used antibody, even though there were big differences in the modus operandi of these antibodies. While the “118” antibody was binding the CD73 molecule without altering its surface expression levels and only inhibited its enzymatic activity, the AD2 antibody caused clustering and internalization [37]. Similarly, the 4G4 antibody did not only inhibit the enzymatic activity of CD73, but also caused shedding of the molecule from LECs and BECs as shown earlier for lymphocytes [38]. Despite this, with the exception of the altered surface expression of CD73 we could not observe any effect of these antibodies on mRNA levels of CD73 nor a difference in their effectiveness of blocking the enzymatic activity of CD73. Interestingly, the enzymatic activity following the antibody blocking was comparable to CD73‐silenced cells, while the surface expression (relative to their controls) remained at higher levels (Supporting Information Fig. S12). Following RNA sequencing of these cells, we could detect some changes due to the treatment with the 4G4 (shedding) antibody. However, only a small number of genes were mildly affected and as we did not observe these alterations with qPCR, it is therefore reasonable to assume that these hits were rather false‐positive than actual altered genes. The fact that we did not observe an altered maturation of moDCs following co‐culture with antibody blocked LECs additionally indicates that the use of CD73‐targeting antibodies does not have far‐reaching effects on the vascular system and thereby the risk of potential negative side or adverse effects is limited. This is especially noteworthy when considering the ongoing clinical trials with CD73‐targeting antibodies (Supporting Information Table S1). In these trials, different CD73‐targeting antibodies such as BMS‐986179, CPI‐006, NZV930, or Oleclumab (MEDI9447) are given either on their own or in combination with other drugs to cancer patients. The main rationale behind this is that blocking of CD73 would prevent the dampening of the immune system and therefore enable the clearance of the cancer, as had been demonstrated in multiple cell and animal studies [11, 12]. The different impact of the silencing/KO and the antibody blocking on the transcriptomics is likely a result of a multitude of factors. First, although two of the used antibodies cause a reduction of CD73 from the cell surface, this reduction is not as comprehensive as with siRNA and it is possible that only after the CD73‐levels drop to a certain threshold transcriptomic alterations occur. Furthermore, by altering the genomic levels of CD73 (NT5E) directly by silencing, also other genes that are associated with (or controlled by) CD73 are affected and a cascade of events can be triggered. In contrast, using CD73‐blocking antibodies does not alter its genomic expression and therefore these secondary effects do not take place. This is also supported by results from our CRISPR/Cas9 KO, where less genomic reduction of CD73 resulted in smaller alterations of other genes. While pinpointing the exact mechanism goes beyond the scope of this study, we can rule out the enzymatic activity as a driving force for the transcriptomic changes as it was comparable in silenced and antibody‐blocked cells.

Nevertheless, it is important to keep in mind the limitations of our study. First, one has to be aware that the antibodies used for modifying CD73 were of murine origin and therefore might react in a somewhat different way than their humanized counterparts. However, this difference is likely of minor importance in our setup as we did not use an in vivo model and therefore do not have the plethora of potential cross‐reactions. Additionally, the source of the DCs for our verification experiments has to be kept in mind. While we have done comprehensive experiments, using monocyte‐derived DCs as well as different skin‐extracted DCs, DCs can, much like macrophages, take on a wide variety of different phenotypes that might affect the final outcome of the experiments. Finally, one also has to be cautious when extrapolating results obtained from mouse models. The used mice were full KOs, leaving the possibility that the absence of CD73 on other tissues also played a role. In addition, in WT mice the expression of CD73 on LECs is lower and more spotty compared to humans [14].

In conclusion, we have shown here that CD73 exerts immune‐modulating effects on the lymphatic vascular system and can thereby dampen the activity of DCs that are in contact with it. Our study is therefore the first work that unravels a function for CD73 in LECs. It shows the involvement of lymphatic CD73 in immune processes, assigning an important role to CD73 in the immune modulating capacities of the lymphatic endothelium. It therefore seems that one reason for the expression of CD73 on afferent lymphatics is to exert a dampening effect on the immune system and on cells migrating in the lymphatic vessels in particular. This dampening seems to prevent immune cells from being fully activated before they reach the LN and by this, it likely improves the overall immune response as those cells are not getting exhausted prematurely. Furthermore, a complete activation that takes place in the LN ensures an optimal interaction of immune cells and a more efficient immune response. Additionally, we could demonstrate that different CD73‐targeting antibodies (1) did not alter the transcriptomics profile of neither the lymphatic nor the blood endothelium and (2) did not alter the maturation of DCs interacting with LECs. We therefore estimate the risk for potential side effects due to unforeseen transcriptomic alterations or secondary effects in the vasculature to be limited in clinical use.

## Materials and methods

### Study design

The objective of this study was to decipher the role of CD73 on afferent lymphatics and determine what possible side effects blocking of CD73 with antibodies may have on the lymphatic and vascular system. For this, primary human endothelial cells from four different 2–6 year old donors were used. CD73 was thereby modified by siRNA and/or epitope specific antibodies and the effect was analyzed by using RNA sequencing. These modifications as well as subsequent verifying and functional experiments took place in a controlled in vitro culturing system. To obtain statistically meaningful results, for all main experiments a minimum of four biological replicas was used. In experiments with small or more variable results, additional samples were processed. In each figure, “n” indicates the number of biological replicas (i.e., the cells were from different donors and/or the experiments were performed on different days); for each main experiment, cells from at least four individuals were used. In the RNA sequencing, genes with zero counts per million (CPM) in at least half of the samples within each group were removed.

### Flow cytometry, sorting, and culture of primary human dermal endothelial cells

Nonspecific binding sites of human cells were blocked with 100 μg/mL human Ig (KIOVIG, Baxter, Helsinki, Finland), while mouse cells were blocked with 0.2% Fc Block and 2% normal mouse serum for 20 min, before incubation with the antibodies for 30 min. Cells were recorded with a LSR Fortessa flow‐cytometer (BD, Helsinki, Finland), followed by analysis with Tree Star's Flowjo v10 (Ashland, USA) software according to the guidelines by Cossarizza et al. [39]. The antibodies used are listed in Supporting Information Table S2. Appropriate isotype controls have been used for all stainings and the instrument adjusted so that isotype controls gave an MFI of 60.

Primary human dermal microvascular endothelial cells from juvenile foreskin (HDMEC, C‐12210) or human dermal LECs from juvenile foreskin (HDLEC, C‐12216) were purchased from PromoCell (Heidelberg, Germany) and cultured in 50% complete MV media and 50% complete MV2 media or 100% MV2 media, respectively (MV medium C‐22020 and MV2 medium C‐22022 from PromoCell, Heidelberg, Germany). For sorting of HDMECs, the cells were labeled with anti‐podoplanin‐PE (#337003, Biolegend, London, United Kingdom) and sorted on a BD FACSAria (BD, Helsinki, Finland) cell sorter into podoplanin‐positive human dermal LECs (Podo+) and podoplanin‐negative human dermal BECs (Podo−) (Supporting Information Fig. S13). Podo+ cells were cultured in MV2 media, while Podo− cells were cultured in MV media. Primary HDMEC cells from four different donors were used.

### Silencing or knockout of CD73 on LECs

The CD73 gene expression in LEC was modulated using the following three models: (1) siRNA pool, (2) single siRNA, and (3) CRISPR/Cas9. Note that 20,000 cells per well of podoplanin+ LECs were plated onto fibronectin (1μg/mL) coated wells of 12‐well plates. Eighteen hours later, the cells were silenced in antibiotics‐free media with the Lipofectamine RNAiMAX reagent (ThermoFisher Scientific, Espoo, Finland). This was done with 15 nM siRNA of three to four pooled siRNA constructs (SMARTpool, ON‐TARGETplus NT5E siRNA, Dharmacon, Lafayette, USA) for CD73 or using ON‐TARGETplus Non‐targeting Control Pool siRNA as a control for 2 hours or 3 days. Alternatively, 15 nM Silencer Select siRNA (s9736) and its negative control #1 have been used (ThermoFisher Scientific, Espoo, Finland) for 3 days.

CRISPR/Cas9 KO was performed according to the manufacturer's instructions for 3 days by using CRISPRMAX Cas9 transfection agent, TrueCut Cas9 Protein v2, Invitrogen TrueGuide modified sgRNA for CD73 (CRISPR1053901_SGM), and TrueGuide sgRNA negative control (non‐targeting 1) (ThermoFisher Scientific, Espoo, Finland).

### qPCR

Sorted cells were collected and stored in RNAprotect (QIAGEN, Helsinki, Finland) at −70°C. This was followed by RNA extraction with the NucleoSpin RNA kit (Macherey‐Nagel, Dueren, Germany) according to the manufacturer's protocol.

For qPCR assays, conversion of RNA to cDNA was done with SuperScript VILO cDNA Synthesis Kit (Thermo Fisher Scientific, Espoo, Finland), followed by TaqMan Gene Expression Assays (Applied Biosystems, Stockholm, Sweden) or UPL‐probe library qPCR on a 7900HT Fast Sequence Detection System (Applied Biosystems) or a QuantStudio3 (Applied BioSystems). The 2^(‐ddCT)^ method with B2M as a control housekeeping gene was used to determine the expression levels.

Used primer/probes are listed in Supporting Information Table S3.

### RNA sequencing and data analysis

Bulk RNA sequencing was used for siRNA‐pool silenced cells and sc‐RNAseq for CRISPR/Cas9‐knock‐down cells. RNA from 104 samples (four different donors, two different cell types, two different time points, and six to eight different treatments) was assayed using RNA sequencing (Supporting Information Fig. S14). The quality of the extracted RNA was verified with Advanced Analytical Fragment Analyzer and the concentration measured with Qubit Fluorometric Quantitation (Life Technologies). High‐quality RNA was used for preparing sequencing libraries according to the Illumina TruSeq mRNA Sample preparation Guide (# 15031047) before these libraries were then sequenced using 50‐bp single‐end sequencing on an Illumina HiSeq‐3000 instrument.

The sequencing data were processed as described previously [40]. Briefly, following a cleaning step for low‐quality, short read‐length and Illumina adapters with Trimmomatic [41], the remaining reads were aligned to the human genome (GRCh38) using STAR [42]. The alignment step was guided by EnsEMBL v82 gene models and involved the use of default 2‐pass per sample parameters, with a modified overhang of the splice junctions of 49. Alignments were sorted and PCR duplicates marked by using Picard tools. The number reads mapped to each genome feature was obtained with SubRead [43]. The Trimmed mean of *M*‐values [44] was then used to normalize expression estimates. Default settings were used with exception of allowing assignments to overlapping genome features. Lowly expressed features were then filtered by removing genes that had zero CPM in at least half the samples within each group.

All statistical analyses were performed with R 3.5.1 [45].

Data were normalized by transforming the raw counts to log2‐CPM (logCPM) with the voom algorithm from package *limma* and subsequently fit with the lmFit function [46]. Considering that observations from different treatments referred to the same donor, the donor effect was adjusted by employing the linear model

$$\;y_{gij}=\mu_{gi}\;+\theta_{gj}+\sigma_{g}\varepsilon_{gij}$$

where $y_{gij}$ is the observed value of gene g corresponding to individual i and treatment j. Parameter $\mu_{gi}$ is the average interaction effect of donor i with gene g, $\theta_{gj}$ is the average interaction effect of treatment j, with gene g, $\sigma_{g}$ is the $\sigma_{g}$ is the SD associated with gene g and $\varepsilon_{gij}$ are standard normally distributed errors. After fitting the linear model, moderated statistics and moderated differential expressions were computed by an empirical Bayes approach using the *eBayes* function from the *limma* package. Gene set enrichment analysis was done with package *EGSEA* [47], containing more than 25 000 gene sets from KEGG [48], MSigDB [49, 50], and GeneSetDB [51]. Finally, a false discovery rate (FDR) adjustment was applied to hypothesis testing results with the *fdrtool* package [52] and tests were marked as significant if the FDR adjusted *p*‐value was less than 0.05.

Selected significantly altered inflammation‐associated genes after 3 days of siCD73 treatment were visualized in a gene‐interaction network. Predictions based on curated gene interactions from the literature were made with the GeneMania [53] plugin in CytoScape 3.6.1 [54].

### Generation and maturation of human monocyte‐derived DCs

Buffy coats were obtained from the Finnish Red Cross Blood Service in Helsinki (permit number 22/2018) and monocytes were extracted by gradient centrifugation (Ficoll‐Paque PLUS, GE Healthcare, Helsinki, Finland) followed by negative bead‐selection with the Monocyte Isolation Kit II (MiltenyiBiotec, Lund, Sweden). Note that 2 × 10^6^ extracted monocytes were cultured in 6‐well plates for 6 days in 3 mL RPMI 1640 with 10% FCS, 4 mM l‐glutamine, 100 U/mL penicillin, and 100 μg/mL streptomycin with the addition of 500 U/mL GM‐CSF (300‐03; Peprotech, London, United Kingdom) and 500 U/mL IL‐4 (204‐IL, R&D Systems, Abingdon, United Kingdom). Fifty percent of media was replaced on day 3. Cells obtained on day 6 were considered immature moDCs, while matured moDCs were generated by adding 500 U/mL IFN‐γ (300‐02; Peprotech) and 100 ng/mL LPS (L‐3024; Sigma, Helsinki, Finland) on day 6.

### Isolation of human skin DCs

After informed consent and permission from the local ethics authorities at the Turku University Hospital (Turku, Finland), human skin from corrective or plastic surgery was collected in the Turku University Hospital. Additional skin samples were also collected in Innsbruck at the Department of Plastic, Reconstructive and Aesthetic Surgery and processed at the Department of Dermatology, Venereology and Allergology of the Medical University Innsbruck (permit “AN 5003 360/5.22 of 15.04.2016”; Innsbruck, Austria). Skin samples were treated for 30 min with either a mix of 100 U/mL penicillin, 100 μg/mL streptomycin, 50 μg/mL gentamicin and 0.25 μg/mL Fungizone or with 50 μg/mL gentamicin alone. Fat was trimmed off and the skin cut into small pieces. These were incubated in either Hank's Balanced Salt Solution (Thermo Fisher Scientific) containing 1 mg/mL Collagenase D (11088866001; Roche, Basel, Switzerland) or RPMI 1640 medium (Lonza, Basel, Switzerland) supplemented with 10% FCS containing 1 mg/mL collagenase IV (Worthington Biochemical Corporation, Lakewood, USA) for 16 h at 37°C. The tissue was then pressed through cell strainers (Corning, Thermo Fisher Scientific) to obtain single cell suspensions.

### Co‐culture of moDCs/skin DCs and HDLECs

Silenced or control‐treated HDLECs were cultured side‐by‐side to confluency in 6‐well plates (Greiner Bio‐One, BioNordika Oy, Helsinki, Finland). The medium was replaced with fresh MV2‐medium containing either 1.5 × 10^6^ moDCs matured for 6 h or 0.5–1 × 10^6^ isolated skin cells. The cells were co‐cultured for 1 day.

### Adhesion assay

siCD73‐silenced and control HDLECs were seeded onto IBIDI chamber slides (μ‐Slide VI, IBIDI, Martinsried, Germany). One day later, either immature or moDCs matured for 6 h with LPS/IFN‐γ were labeled with CFSE (Thermo Fisher Scientific) and added to the slides (5000 cells/lane). After 45 min, nonadherent cells were rinsed away and the remaining cells fixed with paraformaldehyde. Slides were recorded with a total internal reflection microscopy microscope (Carl Zeiss MicroImaging GmbH, Jena, Germany) and analyzed with ImageJ (Fiji, NIH, Bethesda, USA).

### Culture of moDCs with inhibitors of purinergic signaling

On day 6, moDCs were cultured together with DMSO, AMPCP (adenosine 5′‐(α,β‐methylene)diphosphate), alloxanthine, CPCA (5′‐(N‐cyclopropyl)‐carboxamido‐adenosine), or NECA (5′‐N‐ethyl‐carboxamide‐adenosine) for 1 day together with the maturation stimulus. All chemicals were purchased from Sigma and used at a concentration of 15 μM, except alloxanthine (150 μM) and Neca (100 μM).

### Antibody blocking of CD73

CD73 on blood and LECs was blocked by adding azide‐free anti‐CD73 antibodies or respective control antibodies to the culture for either 2 h or 3 days at a concentration of 10 μg/mL. The used antibodies were selected based on their different functions, namely 118 is blocking only the enzymatic activity of CD73 while AD2 is causing clustering and internalization and 4G4 causes shedding of the CD73 [37, 38]. Antibodies are listed in Supporting Information Table S2.

### Enzymatic assays

Enzymatic activities were determined using [2‐^3^H]AMP (Amersham Biosciences/GE Healthcare, Uppsala, Sweden), [2,8‐^3^H]ADP (PerkinElmer, Turku, Finland), and [2,8‐^3^H]ATP (PerkinElmer) as tracer substrates as described previously.

Briefly, 4 mM β‐glyerophosphate (Sigma) was added to the reaction and adenosine triphosphatase (ATPase) and adenosine diphosphatase (ADPase) activities were measured by incubating 2.5 × 10^4^ cells with 300 μM [2,8‐^3^H]ATP or 300 μM [2,8‐^3^H]ADP together with 100 μM diadenosine pentaphosphate (Ap5A; Sigma), respectively, for 45 min at 37°C. Ecto‐5′‐nucleotidase activity was measured by incubating 3 × 10^4^ cells with 50 μM [2‐^3^H]AMP. The adenylate kinase activity was measured by incubating 2.5 × 10^4^ cells with 300 μM [2‐^3^H]AMP and 700 μM γ‐phosphate‐donating ATP for 45 min at 37°C. After the incubation, aliquots were applied onto Alugram G/UV254 plates (Macherey‐Nagel, Düren, Germany) and separated by using TLC with 1‐butanol, 3‐methyl‐1‐butanol, diethylene glycol monoethyl ether, ammonia, and Milli‐Q‐Water (9:6:18:9:15) as solvent. Enzymatic activities were measured by scintillation β‐counting on a Wallac 1409 (PerkinElmer) and expressed as nmol of labeled substrate, which was metabolized by 1 million cells per hour.

### Statistical analysis

Except RNA sequencing data, other data were analyzed with GraphPad Prism 6.02 (Graph‐Pad Software, San Diego, USA). Unless mentioned otherwise, Wilcoxon matched‐pairs signed rank test was used. Values of *p < 0.05 were considered statistically significant (**p < 0.01, ***p < 0.001).

#### scRNA‐seq and data analysis

scRNA‐seq was performed from CRISPR/Cas9 KO and control cells from two different donors. Note that 10 000 cells of each sample were sequenced on a NovaSeq‐6000 sequencer by using the 10xGenomics capture and library prep. Data analysis was performed as previously described [55]. Postprocessing was performed at the Institute for Molecular Medicine Finland (FIMM) using the 10× Genomics Cell Ranger package (v3.1.0). Cell Ranger outputs were analyzed by Seurat (v3.1) for graph‐based clustering and analysis of differentially expressed genes. Data of CD73 KO or control LECs from two donors were merged. Low‐quality cells were removed, the data normalized, and linear transformation was applied on the data by the function of ScaleData. In some analysis, cells with efficient CD73 knock‐down of CD73 KO LECs were selected (less than 0.5 of CD73 expression level) and used for further analysis. Differentially expressed genes between CD73 KO and control LECs were analyzed by the function of “FindMarkers” and DEseq2 [56] was used for the differential expression test. These data will be reported in more detail in a separate study.

### Animals

Male (for footpad‐injections and FITC‐painting) and female (for microscopy) pathogen‐free mice of a CD73^−/−^ or a control C57BL/6 genotype were used at an age of 2–3 months (Animal license numbers 3791/04.10.03/2011, 5587/04.10.07/2014, and 5762/04.10.07/2017). The animals had access to food and water ad libitum and were bred and housed at the Central Animal Laboratory at the University of Turku. This was done following official guidelines on the care and use of laboratory animals and in adherence to the Finnish Act on Animal Experimentation (62/2006). The Finnish Animal Ethics Committee approved the carried out experiments that followed the 3R guidelines.

### FITC ear painting and FACS staining

Mice were anesthetized with isoflurane before 15 μL of a FITC solution (5 mg/mL FITC, F7250; Sigma) in a 1:1 acetone/dibutylphtalate (Thermo Fisher Scientific/Sigma) solution was applied to the dorsal side of each ear. The painted animals were sacrificed after 2 days and the draining LNs were collected (cervical and as control axial/inguinal).

Cells were blocked for 30 min on ice with FC‐block before staining for 30 min with fluorescent antibodies (Supporting Information Table S2, gating in Supporting Information Fig. S15).

### Footpad injections

After being anesthetized, mice were injected with 25 μL (50μg) of ovalbumin (F5503; Sigma) diluted 1:1 in PBS and incomplete Freud's adjuvant (F5506; Sigma) into their footpad. One day later, animals were sacrificed and the draining LNs were collected.

### Extraction of cells from LNs

The LNs were torn apart with forceps and placed in 2 mL extraction medium (RPMI with 2% Hepes and 2% FCS) containing 1 mg Collagenase D (11088866001; Roche) and 0.1 mg DNAse 1 (10104159001; Roche). After incubating at +37°C on a shaker for 30 min, 200 μL 0.1M EDTA was added for 5 min to stop the reaction. The LNs were then grinded through a metal mesh and the solution filtered to obtain a single cell solution.

### Oxazolone model

Two percent oxazolone solution (in 4:1 acetone:olive oil) was applied to the abdominal skin and paws of the mice. After 5 days, animals were challenged by applying a 1% oxazolone solution to their ears and flank skin. Animals were sacrificed 24 h later and tissues were embedded in OCT compound.

### Staining of frozen sections, microscopy, and quantification

Sections were acetone fixed and blocked with 2% BSA for 30 min before being stained with antibodies overnight at +4°C. PBS‐washed slides were then incubated with secondary antibodies for 2 h at room temperature and mounted with Prolong Gold Antifade Mountant (Thermo Fisher Scientific). Slides were recorded with a Zeiss LSM880 microscope and quantified using ImageJ (Fiji, NIH).

## Conflict of interest

S. J. and M. S. own stocks of Faron Pharmaceuticals. The other authors have no additional commercial and financial interests.

**Abbreviations**
BECs: blood endothelial cells
LECs: lymphatic endothelial cells
moDCs: monocyte‐derived DCs

## Supporting information

Supporting Information

## Data Availability

The RNA‐sequencing data are available at the NCBI Gene Expression Omnibus (GEO) database with the accession number GSE131047 (https://www.ncbi.nlm.nih.gov/geo/query/acc.cgi?acc=GSE131047)
