# Supplementary material for: CD73 contributes to anti‐inflammatory properties of afferent lymphatic endothelial cells in humans and mice
Source: Eur J Immunol. 2020 Oct 29;51(1):231–46. doi: 10.1002/eji.201948432 (PMC7821194; doi:10.1002/eji.201948432)
Supplement: Supplementary file 1 — Supporting Information [file EJI-51-231-s001.doc]

**Supplemental Material**

**Supplemental Figures**

**
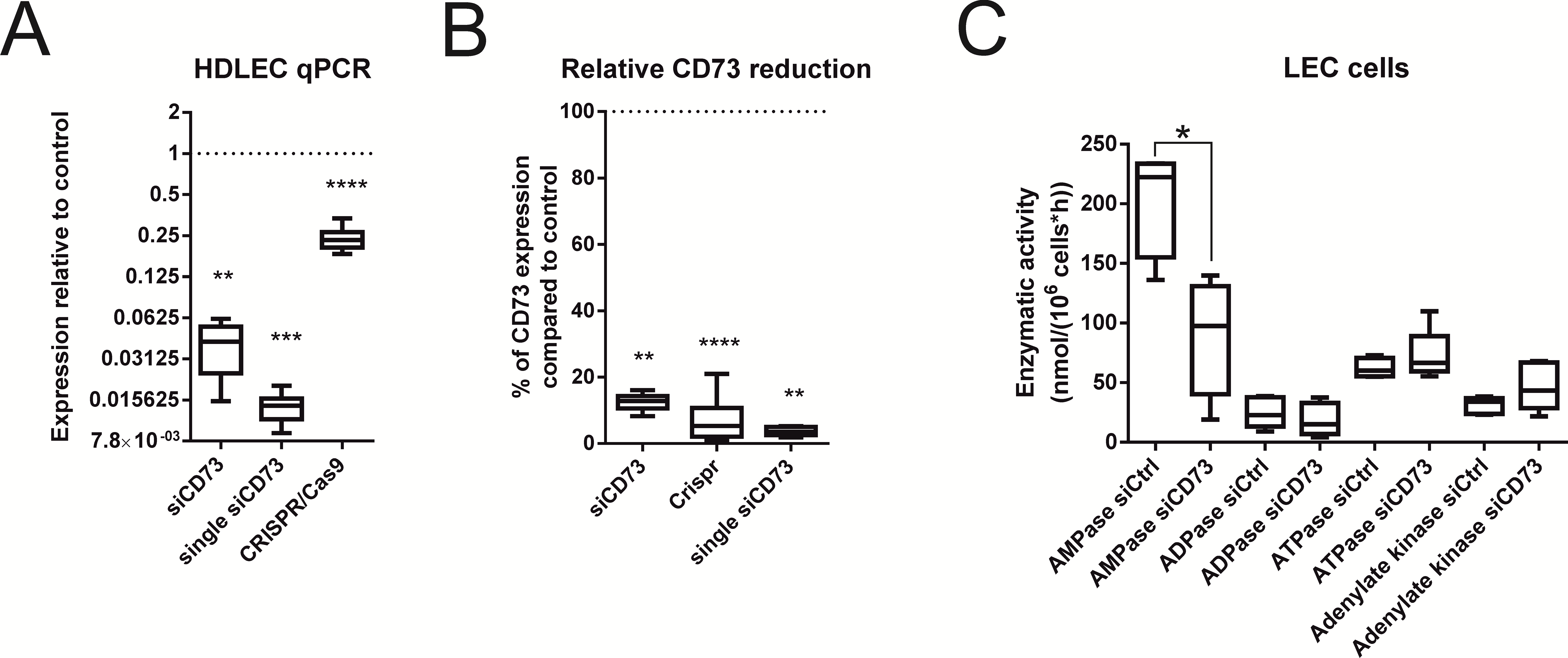
**

**Supporting Information Figure 1: knockdown or siCD73 treatment of LECs strongly reduces CD73 expression**

**(A)** Gene expression of CD73 (NT5E) after silencing or knockdown determined by qPCR. The data are from three to four independent experiments with two to three different biological donors, analyzed with Mann-Whitney U test. **(B)** Surface expression of CD73 in control and CD73-silenced LECs measured by flow-cytometry. The data are from two to five independent experiments with two to three different biological donors, analyzed with Mann Whitney U test. **(C)** Enzymatic activity in control- and CD73-silenced LECs for AMPase (CD73), ADPase, ATPase and Adenylate kinase. The data are from two independent experiments with two to three different biological donors (n=4-5), analyzed with Mann Whitney U test. * indicates P<0.05, ** indicates P<0.01, *** indicates P<0.001, **** indicates P<0.0001.

**
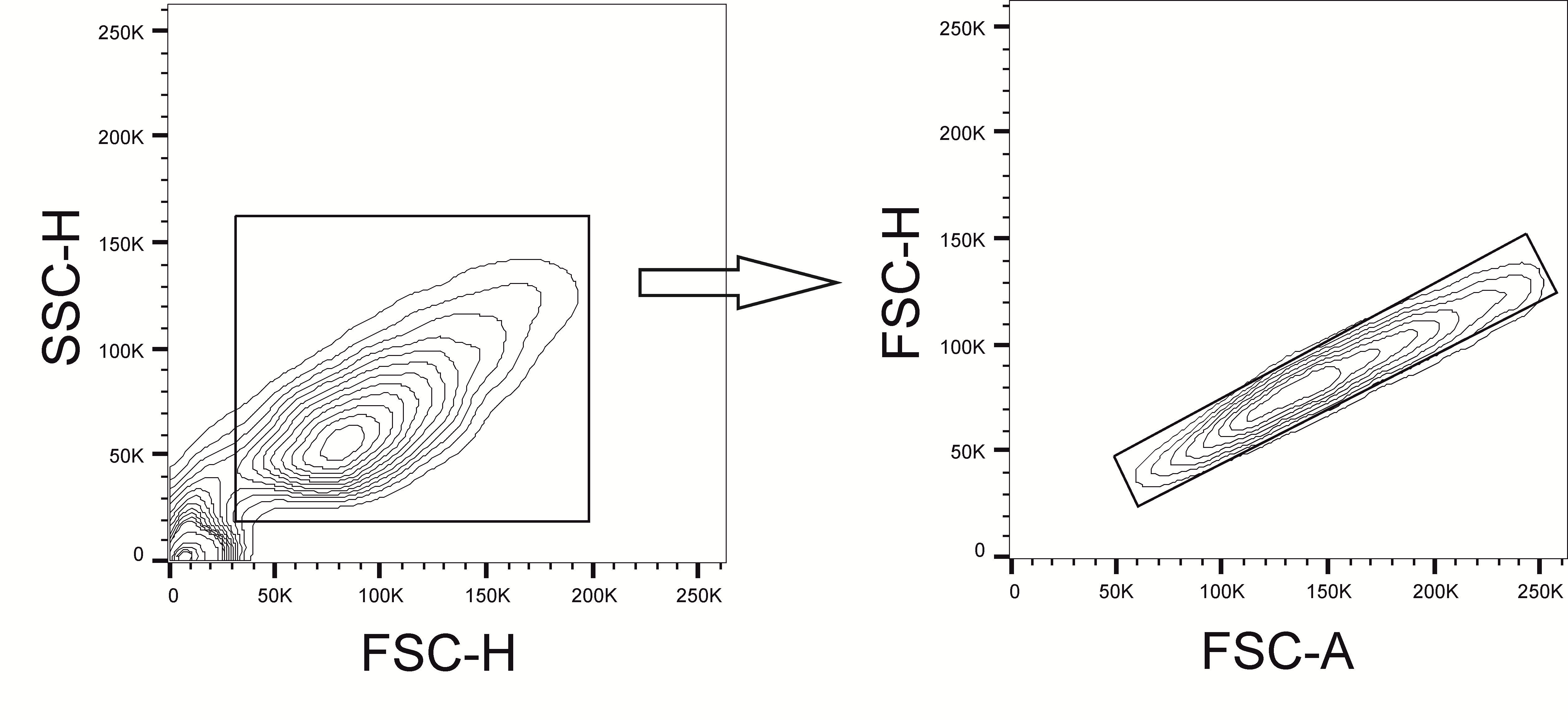
**

**Supporting Information Figure 2: Flow-cytometry gating for human endothelial cells**

Human endothelial cells were first gated according to FSC/SSC before excluding doublets.

**
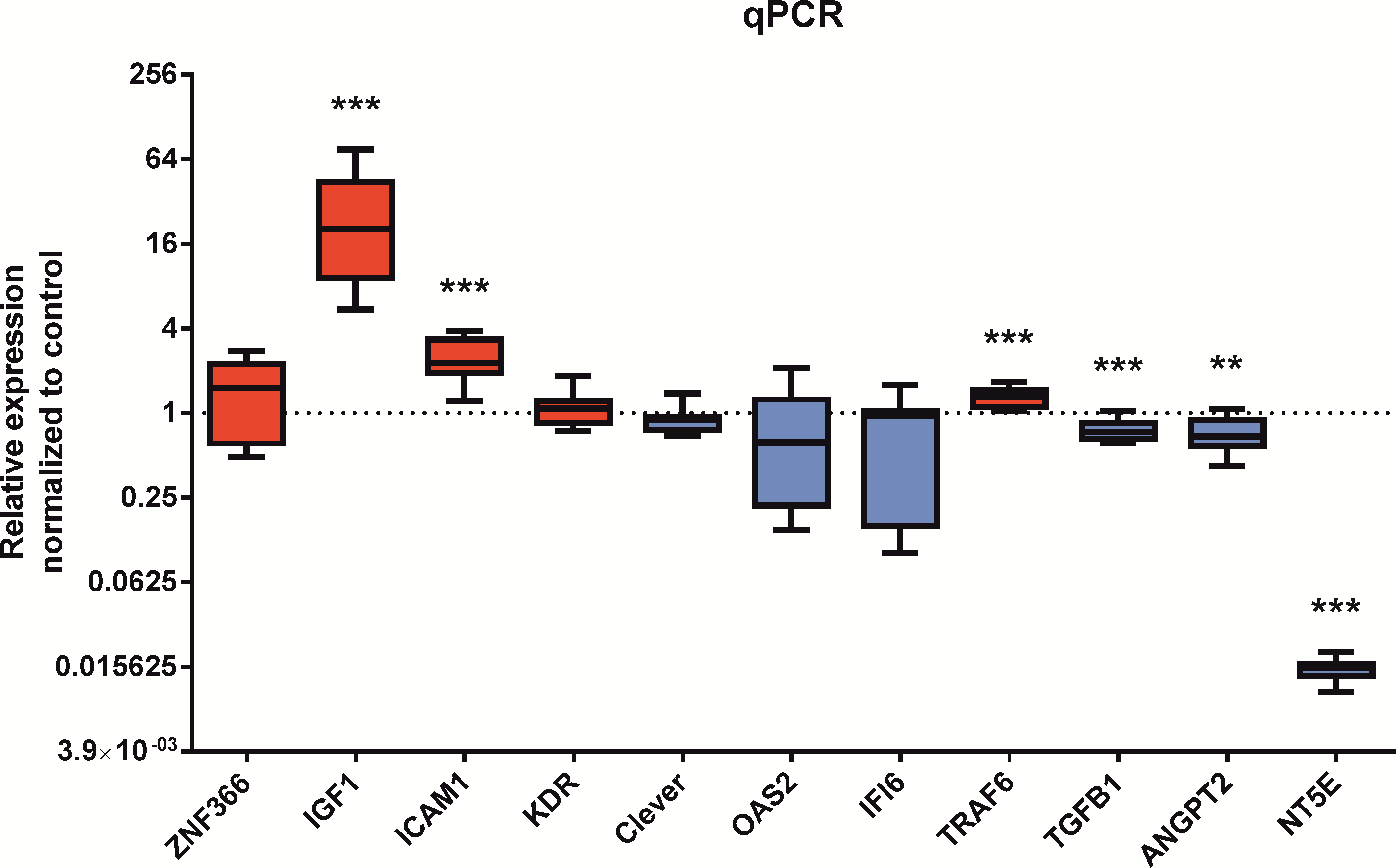
**

**Supporting Information Figure 3: qPCR results showing gene changes after altering CD73 with a single siRNA**

qPCR verification of important RNA-seq hits shown as fold changes of single siCD73-treated LECs compared to non-targeted control, analyzed with Wilcoxon matched-pairs signed rank test. Upregulated genes are marked in red, downregulated genes in blue. * indicates P<0.05, ** indicates P<0.01, *** indicates P<0.001.

**
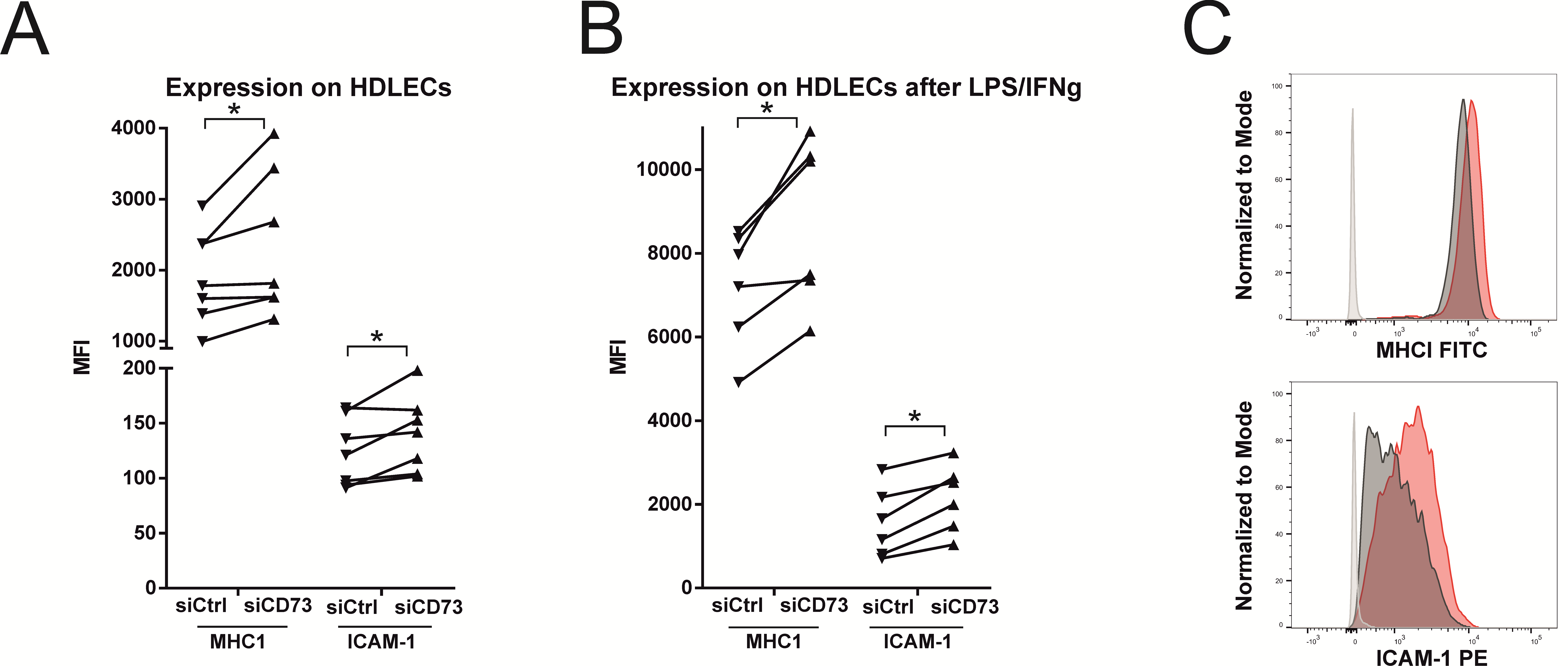
**

**Supporting Information Figure 4: MHCI and ICAM-1 are elevated in CD73-silenced LECs**

**(A)** Median fluorescence intensity (MFI) values of MHCI and ICAM-1 on LECs after siCD73 treatment and **(B)** after additional LPS/IFN-g exposure with representative histograms **(C)** depicting MHCI and ICAM-1 expression (red=siCD73 LECs, dark grey=siCtrl LECs, light grey=Isotype control). Data were obtained with flow cytometry from two to three independent experiments with two, four and one different biological donor(s) and have been analyzed with Wilcoxon matched-pairs signed rank test. * indicates P<0.05


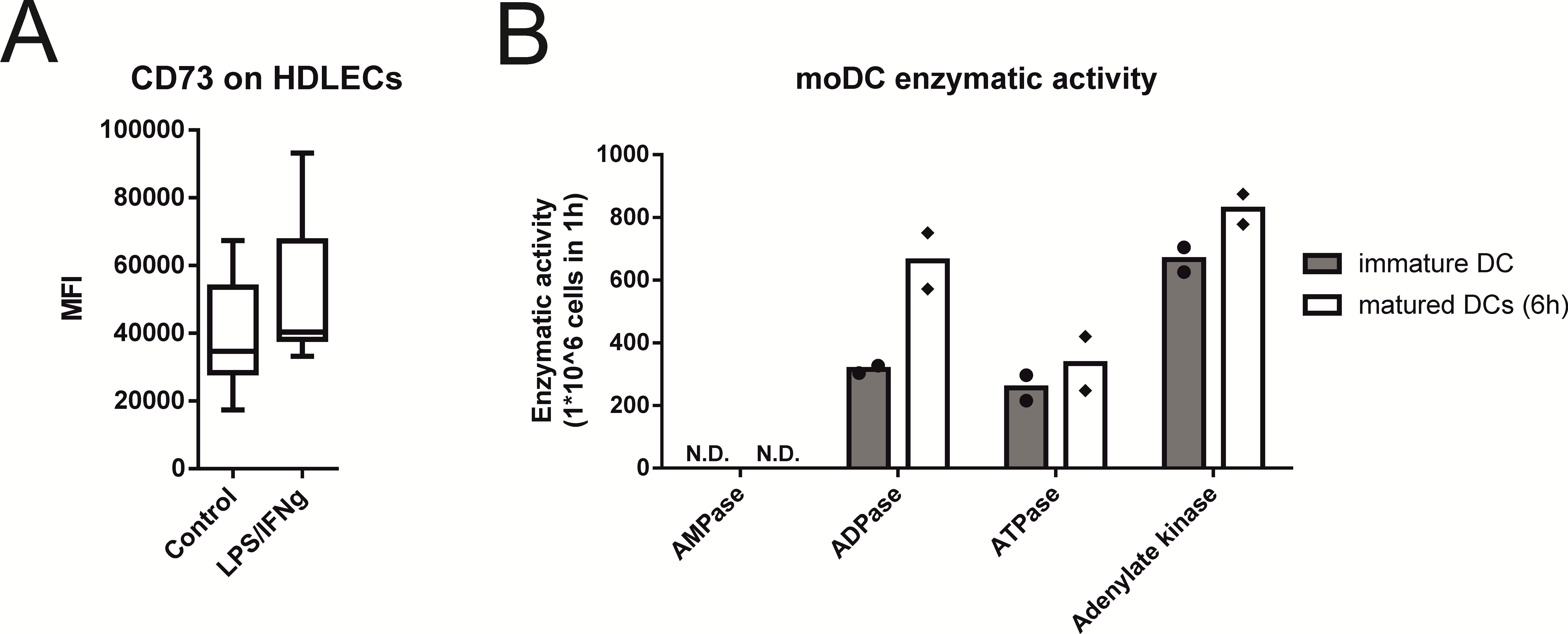


**Supporting Information Figure 5: Effects of LPS/IFN-g on LECs and DCs**

**(A**) Increase in CD73 expression following LPS/IFN-g treatment. Median fluorescent intensity values from flow-cytometry (PE-CF594) of control and LPS/IFN-g treated LECs. The data are from 6 independent experiments with one to three different biological samples (n=13). **(B)** moDCs have no AMPase (CD73) activity. Enzymatic activities of naïve and matured moDCs for AMPase, ADPase, ATPase and Adenylate kinase. Data are from one experiments with two different biological donors. N.D. = not detectable.

**
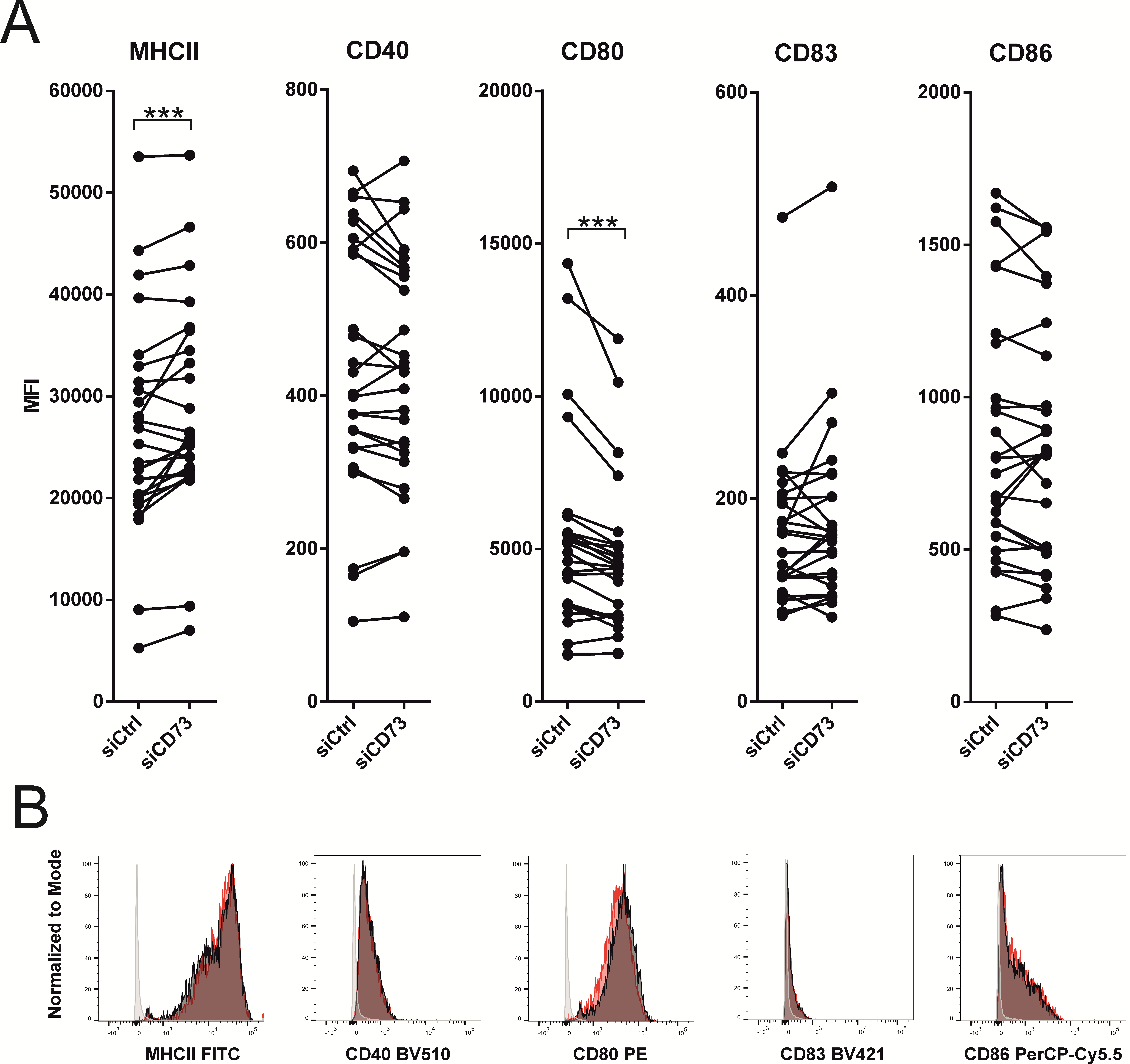
**

**Supporting Information Figure 6: Maturation markers of moDCs are altered after co-culture with CD73-silenced LECs**

**(A)** MFI values obtained from flow cytometry of moDC maturation markers following co-culture with siCD73 treated LECs and their controls. The data are from 17 independent experiments with one to two different biological donors and have been analyzed with Wilcoxon matched-pairs signed rank test. *** indicates P<0.001. **(B)** Representative histograms depicting MHCII, CD40, CD80, CD83 and CD86 expression in moDCs after co-culture (red=siCD73 LECs, dark grey=siCtrl LECs, light grey=Isotype control).


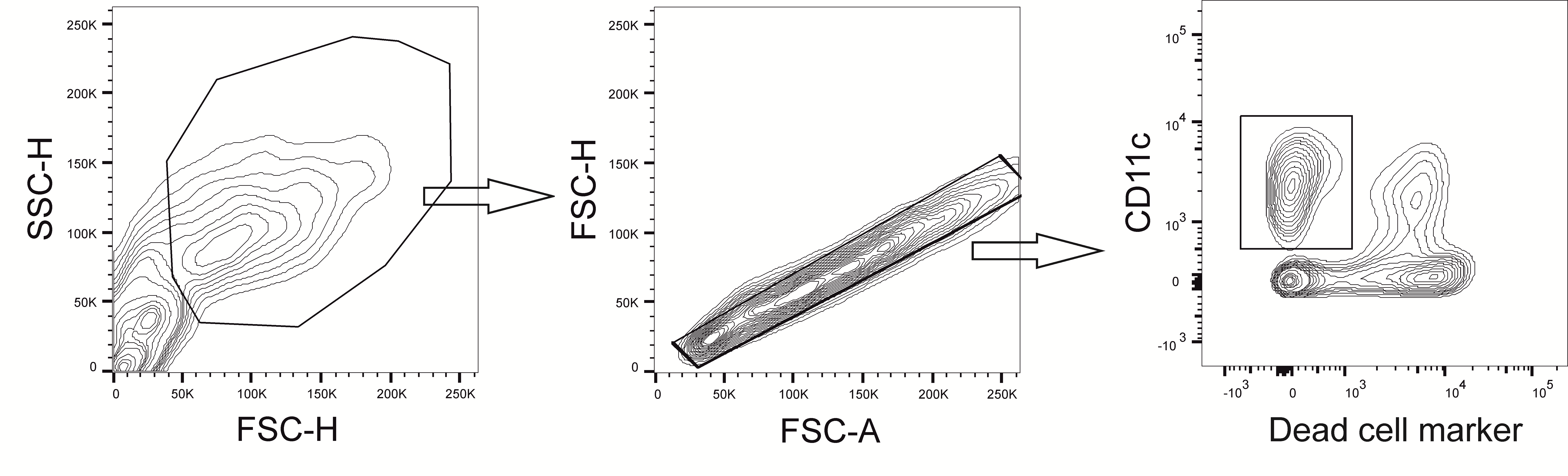


**Supporting Information Figure 7: Flow-cytometry gating strategy for DCs after co-culture with LECs**

Following the co-culture of moDCs and LECs, DCs were gated according to FSC/SSC before excluding doublets and gating on viable CD11c cells.


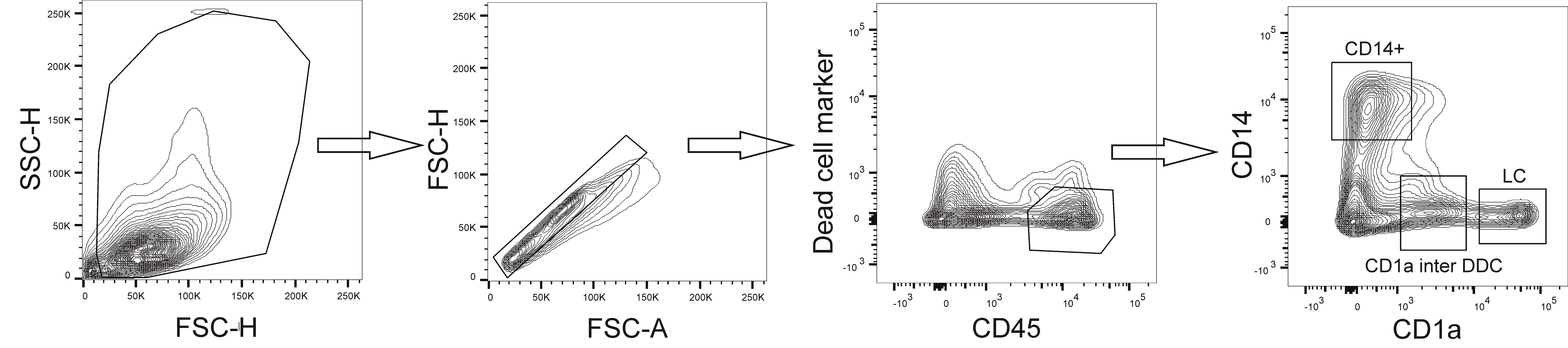


**Supporting Information Figure 8: Flow-cytometry gating strategy for skin-derived DCs**

Human skin-derived cell suspensions were pre-gated according to FSC/SSC and doublets were excluded. Viable leukocytes were sub-gated with CD45 and the Dead-cell-marker. CD14 and CD1a define the three DC subsets: CD1a^+++^/CD14^-^ epidermal Langerhans cells, CD1a^+^/CD14^-^ dermal DCs and CD1a^-^/CD14^+^ dermal monocytic cells. CD40, CD80, CD83 and MHCII were then determined on these populations.

**

**

**Supporting Information Figure 9: Maturation markers of skin-derived immune-cells are altered after co-culture with CD73-silenced LECs**

MFI values and representative histograms of skin-DC maturation markers **(A)** MHCII, **(B)** CD40, **(C)** CD80 and **(D)** CD83 following co-culture with siCD73 treated LECs and their controls. The data are from six independent experiments with one to three different biological donors and have been analyzed with Wilcoxon matched-pairs signed rank test. * indicates P<0.05 , ** indicates P<0.01, *** indicates P<0.001. red=siCD73 LECs, dark grey=siCtrl LECs, light grey=Isotype control.


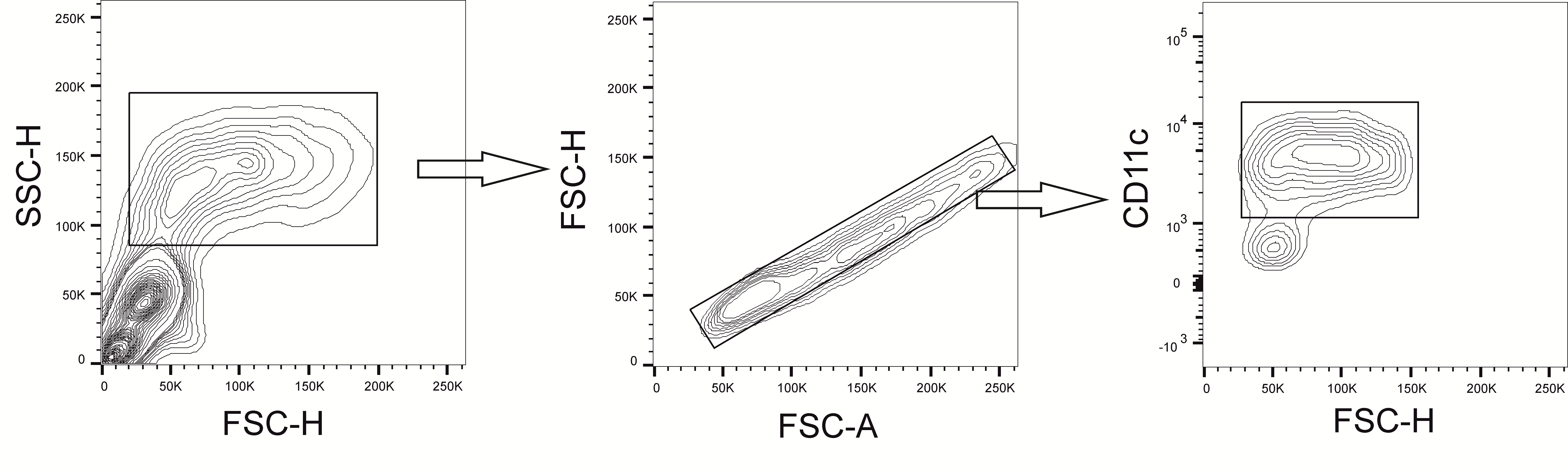


**Supporting Information Figure 10: Flow-cytometry gating strategy for moDCs**

moDCs cultured alone were gated on FSC/SSC before excluding doublets and gating on CD11c positive cells.

**
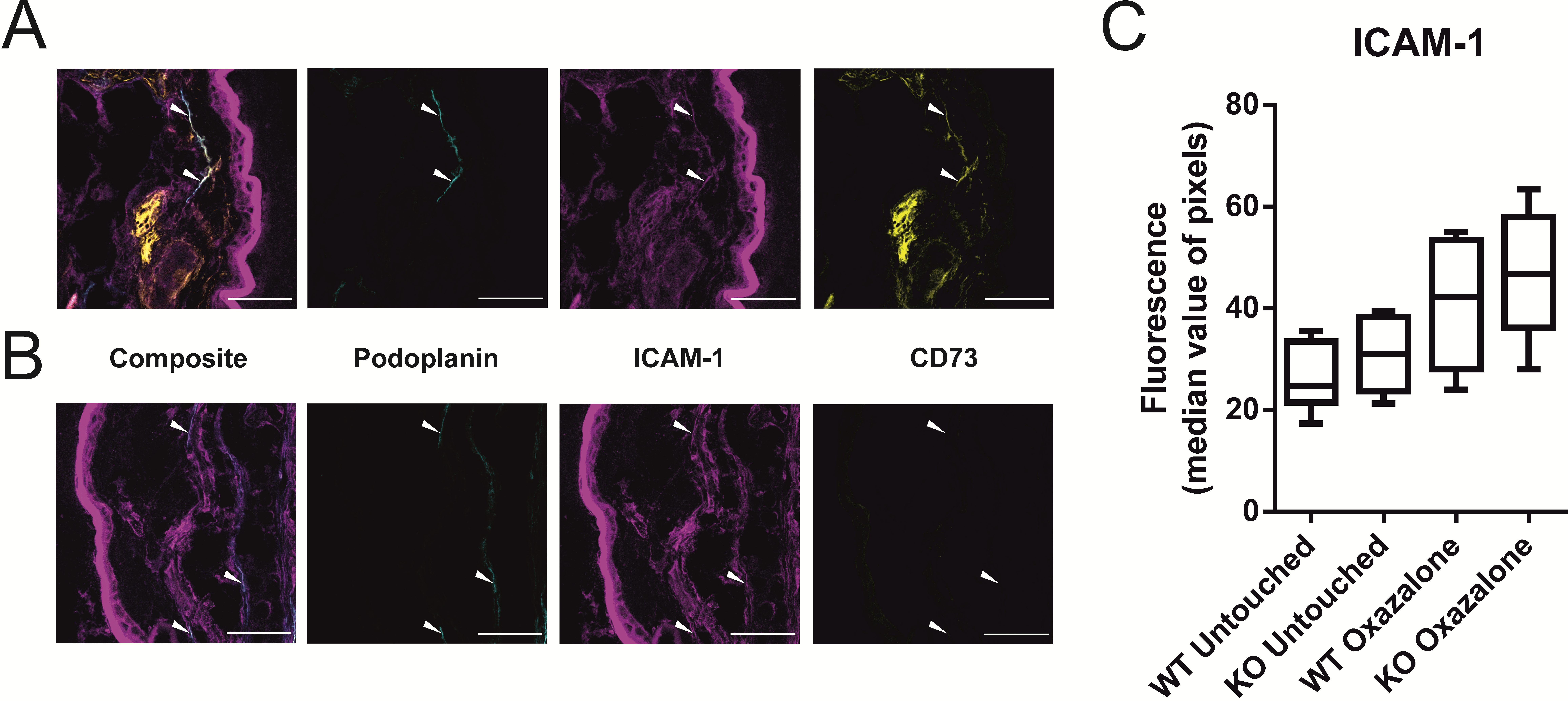
**

**Supporting Information Figure 11: ICAM-1 levels on lymphatic vessels from CD73 KO and WT mice**

ICAM-1 (CD54) levels on Podoplanin-positive lymphatic vessels were determined in skin sections from untreated and Oxazolone challenged KO and WT animals. Representative stainings from **(A)** WT and **(B)** KO animals are shown with Podoplanin (cyan), ICAM-1 (magenta) and CD73 (yellow) following Oxazolone challenge. **(C)** Quantification of ICAM-1 levels in untreated and Oxazolone-challenged WT and KO animals. White arrowheads indicate examples of lymphatic vessels. Used magnification was 20x and the scale bar length is 50 µm. The data are from one experiment with three different biological donors for the “untouched” results and from two independent experiments with four to five different biological donors for the Oxazolone results.


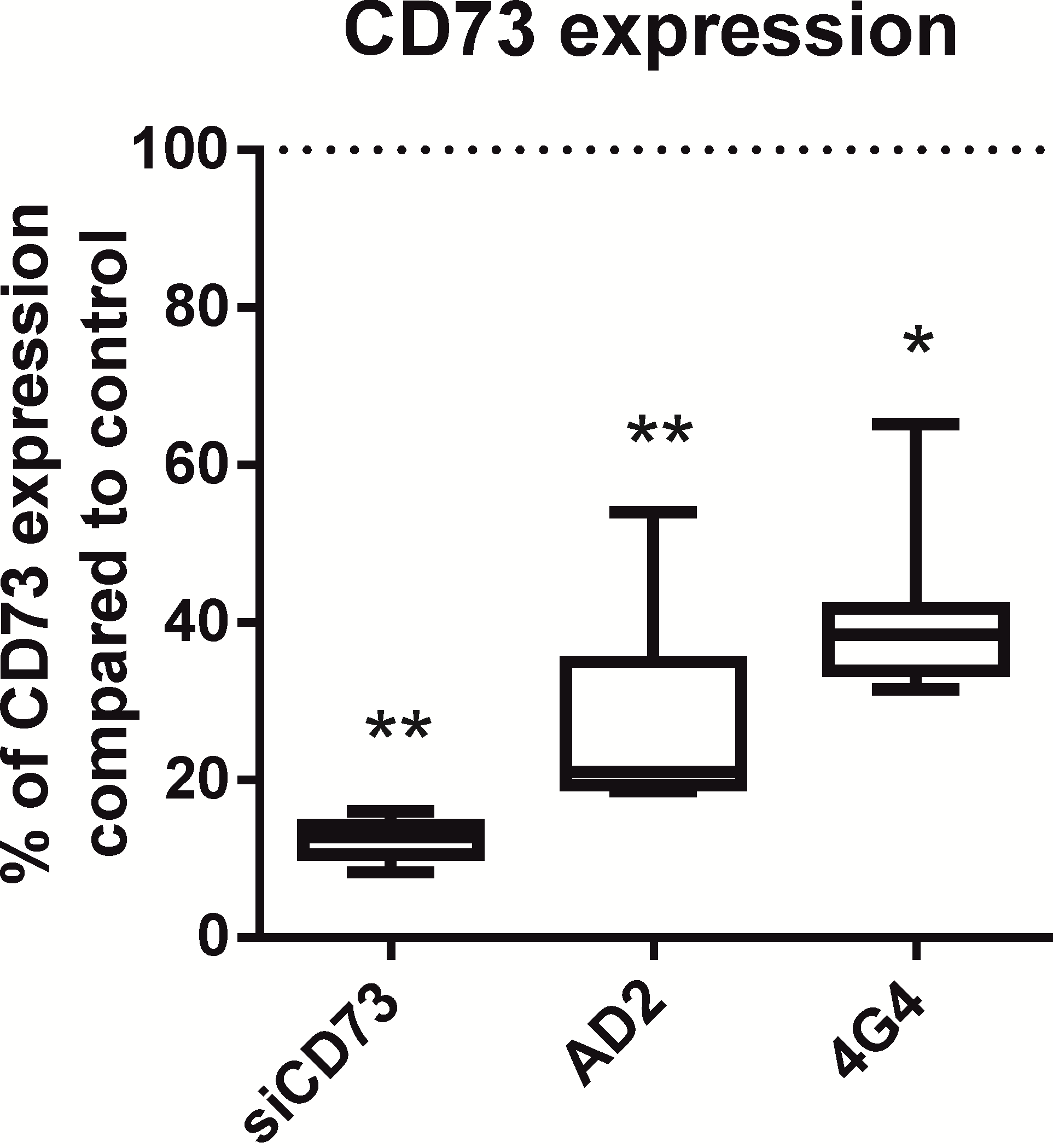


**Supporting Information Figure 12: CD73 protein expression after silencing or blocking CD73**

Surface expression of CD73 in control and CD73-silenced or antibody-treated LECs measured by flow-cytometry. The data are from two to five independent experiments with two to three different biological donors, analyzed with Mann Whitney U test. * indicates P<0.05 , ** indicates P<0.01.

**
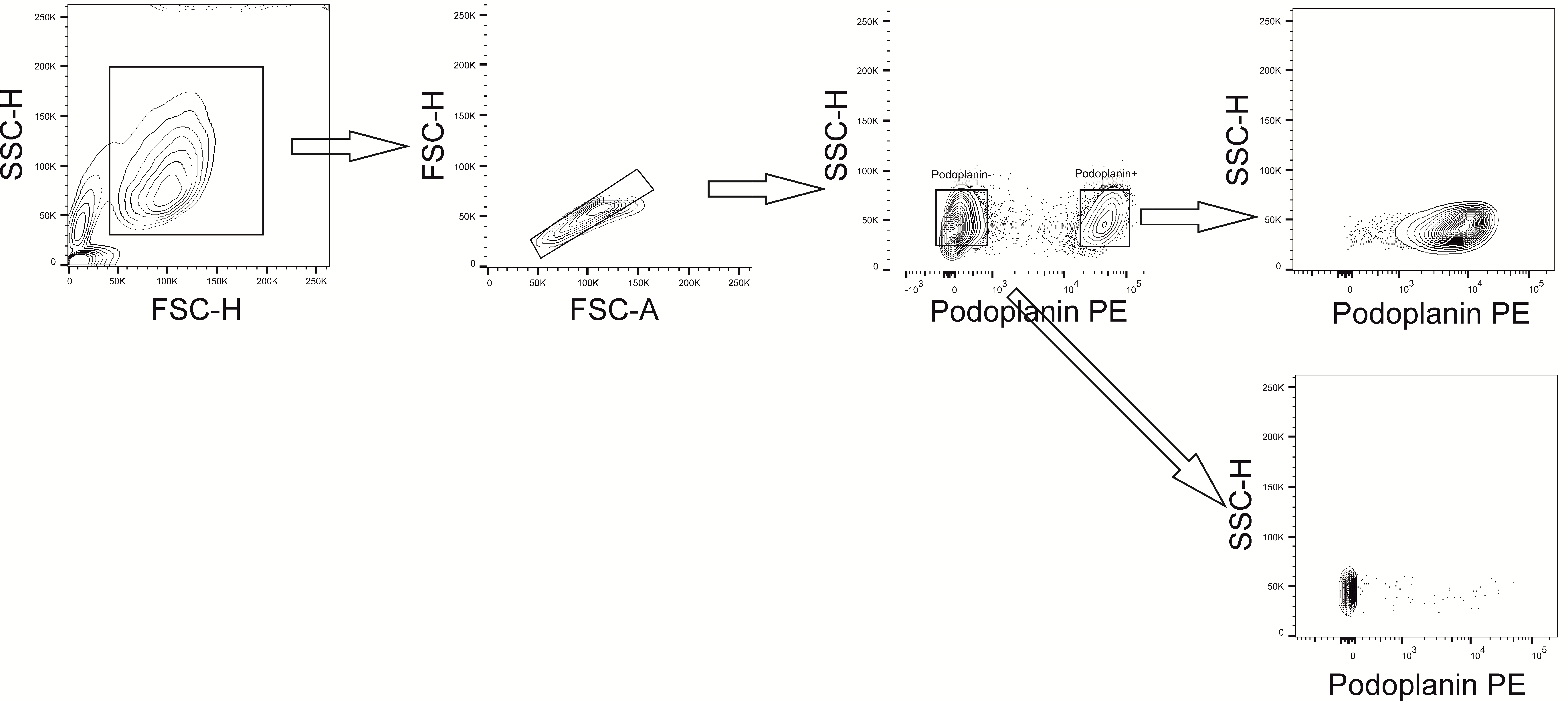
**

**Supporting Information Figure 13: Flow-cytometry gating strategy for sorting of HDMECs**

HDMECs were pre-gated according to FSC/SSC and single cells were sorted according to their Podoplanin expression into Podo+ and Podo- cells. The average purity after sorting was >97%.

**Supporting Information Figure 14: Experimental setup**

Overview depicting the different samples and treatments used for the RNA-sequencing.

**
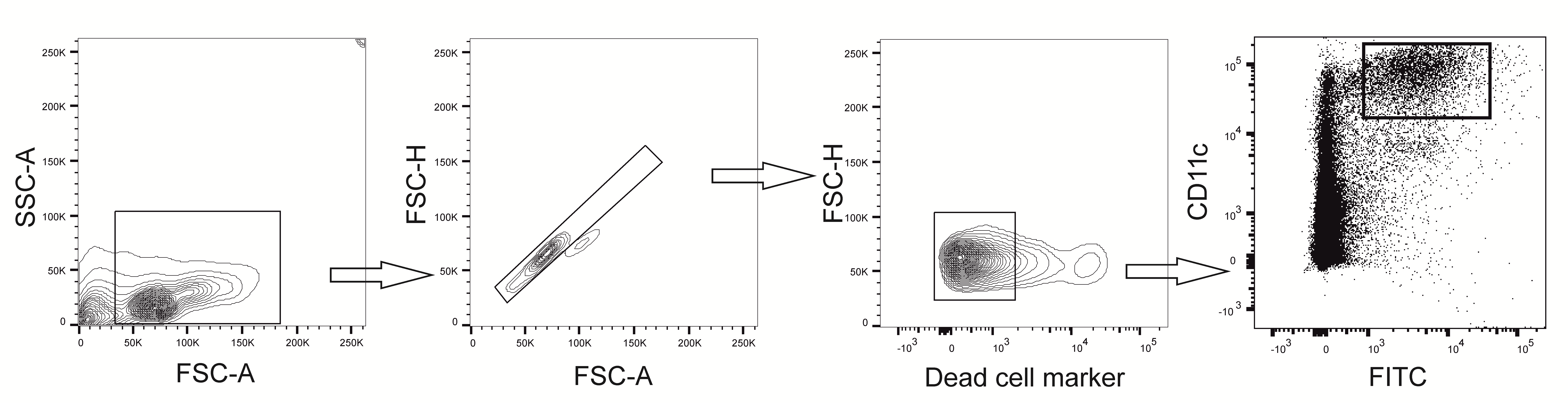
Supporting Information Figure 15: Flow-cytometry gating strategy for mouse cells**

Extracted mouse cells were first gated according to FSC/SSC before excluding doublets and dead cells. For FITC painting experiments, they were then gated for CD11c and FITC.

**Supplemental tables**

| **Study identifier** | **Study name and URL** |
| --- | --- |
| Identifier NCT02754141 | An Investigational Immuno-therapy Study of Experimental Medication BMS-986179 Given Alone and in Combination With Nivolumab. 2016. Available at: https://clinicaltrials.gov/ct2/show/NCT02754141 [Accessed March 15, 2019]. |
| Identifier NCT02503774 | MEDI9447 Alone and in Combination With MEDI4736 in Adult Subjects With Select Advanced Solid Tumors. 2015. Available at: https://clinicaltrials.gov/ct2/show/NCT02503774 [Accessed March 15, 2019]. |
| Identifier NCT03267589 | Trial in Patients With Relapsed Ovarian Cancer. 2018. Available at: https://clinicaltrials.gov/ct2/show/NCT03267589 [Accessed March 15, 2019]. |
| Identifier NCT03736473 | A Phase I Study of MEDI9447 (Oleclumab) in Japanese Patients. 2018. Available at: https://clinicaltrials.gov/ct2/show/NCT03736473 [Accessed March 15, 2019]. |
| Identifier NCT03334617 | Phase II Umbrella Study of Novel Anti-cancer Agents in Patients With NSCLC Who Progressed on an Anti-PD-1/PD-L1 Containing Therapy. (HUDSON). 2017. Available at: https://clinicaltrials.gov/ct2/show/NCT03334617 [Accessed March 15, 2019]. |
| Identifier NCT03549000 | A Phase I/Ib Study of NZV930 Alone and in Combination With PDR001 and /or NIR178 in Patients With Advanced Malignancies. 2018. Available at: https://clinicaltrials.gov/ct2/show/NCT03549000 [Accessed August 30, 2019]. |
| Identifier NCT03454451 | CPI-006 Alone and in Combination With CPI-444 and With Pembrolizumab for Patients With Advanced Cancers. 2018. Available at: https://clinicaltrials.gov/ct2/show/NCT03454451 [Accessed August 30, 2019]. |

**Supporting Information Table 1.** Clinical studies using CD73 modulating agents as listed on ClinicalTrials.gov (Bethesda (MD): National Library of Medicine (US))

| **Antibody** | **Target Species** | **Isotype** | **Product #** |
| --- | --- | --- | --- |
| CD1 PE | Human | Mouse IgG2a | LS Bio LS-C196193 |
| CD1a AF700 | Human | Mouse IgG1 | Biolegend 300120 |
| CD1a PerCP-Cy5.5 | Human | Mouse IgG1 | Biolegend 300130 |
| CD11c APC | Human | Mouse IgG1 | BD559877 |
| CD14 APC | Human | Mouse IgG2a | BD 555399 |
| CD14 PE | Human | Mouse IgG1 | Biolegend 325606 |
| CD31 FITC | Human | Mouse IgG1 | Beckman Coulter IM1431U |
| CD40 APC | Human | Mouse IgG1 | Biolegend 334310 |
| CD45 BV421 | Human | Mouse IgG1 | BD 563879 |
| CD40 BV510 | Human | Mouse IgG1 | BD 563456 |
| CD45 BV510 | Human | Mouse IgG1 | Biolegend 304036 |
| CD45 PE | Human | Mouse IgG1 | BD 555483 |
| CD73 (118) | Human | Mouse IgG2b | in-house |
| CD73 (4G4) | Human | Mouse IgG1 | in-house |
| CD73 (AD2) | Human | Mouse IgG1 | BD 550256 |
| CD73 PE-CF594 | Human | Mouse IgG1 | BD 562817 |
| CD73 PerCP-Cy5.5 | Human | Mouse IgG1 | BD 561260 |
| CD80 PE | Human | Mouse IgG1 | BD 557227 |
| CD83 BV421 | Human | Mouse IgG1 | BD 562630 |
| CD83 BV421 | Human | Mouse IgG1 | Biolegend 305324 |
| CD86 PerCP-Cy5.5 | Human | Mouse IgG1 | BD 561129 |
| HLA Class I FITC | Human | Mouse IgG2a | Sigma F5662 |
| HLA-DP,-DQ,-DR FITC | Human | Mouse IgG2a | BD 555558 |
| HLA-DR PE-Cy7 | Human | Mouse IgG2a | Biolegend 307616 |
| ICAM-1 | Human | Rabbit IgG | Santa Cruz sc-7891 |
| Podoplanin PE | Human | Rat IgG2a | Biolegend 337004 |
| IgG1 | - | Mouse | BD 555746 |
| IgG1 AF700 | - | Mouse | BD 557882 |
| IgG1 APC | - | Mouse | R&D IC002A |
| IgG1 APC | - | Mouse | Biolegend 400120 |
| IgG1 BV421 | - | Mouse | Biolegend 400157 |
| IgG1 BV421 | - | Mouse | Biolegend 400158 |
| IgG1 BV510 | - | Mouse | BD 562946 |
| IgG1 BV510 | - | Mouse | Biolegend 400172 |
| IgG1 FITC | - | Mouse | BD 554679 |
| IgG1 PE | - | Mouse | BD 555749 |
| IgG1 PE | - | Mouse | Biolegend 400114 |
| IgG1 PE-CF594 | - | Mouse | BD 562292 |
| IgG1 PerCP-Cy5.5 | - | Mouse | BD 552834 |
| IgG1 PerCP-Cy5.5 | - | Mouse | Biolegend 400150 |
| IgG2a APC | - | Mouse | BD 340473 |
| IgG2a FITC | - | Mouse | BD 553456 |
| IgG2a PE-Cy7 | - | Mouse | Biolegend 400232 |
| IgG2b | - | Mouse | Biolegend 401212 |
| Fixable Viability Dye eFluor 780 | - | - | eBioscience 65-0865-18 |
| normal rabbit IgG | - | Rabbit | Santa Cruz sc-3888 |
| CD11c BV421 | Mouse | Hamster IgG1 | BD 562919 |
| CD11c PerCP-Cy5.5 | Mouse | Hamster IgG1 | BD 550763 |
| CD40 APC | Mouse | Rat IgG2a | BD 560720 |
| CD40 PE-CF594 | Mouse | Rat IgG2a | BD 562302 |
| CD54 AF488 | Mouse | Rat IgG2b | Southern Biotech 1701-30 |
| CD73 (rNu-9) | Rat | Rabbit IgG | Provided by Prof. Jean Sevigny, Laval University, Quebec, Canada (http://ectonucleotidases-ab.com) |
| CD80 PerCP-Cy5.5 | Mouse | Hamster IgG2 | BD 560562 |
| CD86 APC | Mouse | Rat IgG2a | BD 560720 |
| CD86 PE | Mouse | Rat IgG2a | BD 553930 |
| I-A/I-E (MHC II) BV510 | Mouse | Rat IgG2b | Biolegend 400646 |
| I-A/I-E (MHC II) FITC | Mouse | Mouse IgG2a | BD 553456 |
| Podoplanin APC | Mouse | Hamster IgG | Biolegend 127410 |
| IgG1 BV421 | - | Hamster | BD 562919 |
| IgG1 PerCP-Cy5.5 | - | Hamster | BD 550763 |
| IgG2 PerCP-Cy5.5 | - | Hamster | BD 560562 |
| IgG2a APC | - | Rat | BD 560720 |
| IgG2a PE | - | Rat | BD 553930 |
| IgG2a PE | - | Rat | eBioscience 12-4321-81a |
| IgG2a PE-CF594 | - | Rat | BD 562302 |
| IgG2b BV510 | - | Rat | Biolegend 400646 |
| Fc Block | Mouse | Rat IgG2b | BD 553142 |
| anti IgG AF488 | Rat | Donkey | Invitrogen A21208 |
| anti IgG AF546 | Rabbit | Goat | Invitrogen A11035 |
| anti IgG1 PE | Mouse | Goat IgG | Invitrogen A21123 |
| anti IgG2b PE | Mouse | Goat IgG | Southern Biotech 1090-09 |

**Supporting Information Table 2.** Antibodies used in this study.

| **Primer/Probe** | **Target Species** | **Product #** |
| --- | --- | --- |
| ACTB | Human | Hs99999903_m1 |
| ADORA1 | Human | Hs00181231_m1 |
| ADORA2A | Human | Hs00169123_m1 |
| ADORA2B | Human | Hs00386497_m1 |
| ADORA3 | Human | Hs00181232_m1 |
| ANGPT2 | Human | Hs00169867_m1 |
| B2M | Human | Hs99999907_m1 |
| BST2 | Human | Hs00171636_m1 |
| CD69 | Human | Hs00934033_m1 |
| CLEVER (UPL-probe #74) | Human | left: cac atg tgc caa gaa gat cc |
|  |  | right: cac agc gtg cca aag aaa c |
| ELOVL6 | Human | Hs00907564_m1 |
| EPSTI1 | Human | Hs01566789_m1 |
| ERG | Human | Hs01554629_m1 |
| HES1 | Human | Hs00172878_m1 |
| ICAM1 | Human | Hs00164932_m1 |
| IFI6 | Human | Hs00242571_m1 |
| IGF1 | Human | Hs01547656_m1 |
| IL6R | Human | Hs01075666_m1 |
| KDR | Human | Hs00911700_m1 |
| MX1 | Human | Hs00895608_m1 |
| NT5E | Human | Hs00159686_m1 |
| OAS2 | Human | Hs00942643_m1 |
| OGDH | Human | Hs01081865_m1 |
| S1PR1 | Human | Hs05021992_s1 |
| TGFB1 | Human | Hs00998133_m1 |
| TRAF6 | Human | Hs00939742_g1 |
| ZNF366 | Human | Hs00403536_m1 |

**Supporting Information Table 3.** qPCR primers and probes used in this study.
